# Sampling and curation of rove beetles (Insecta, Coleoptera, Staphylinidae) for comprehensive and DNA-grade collections to enhance biodiversity exploration in Northern Eurasia

**DOI:** 10.3897/BDJ.10.e96080

**Published:** 2022-12-19

**Authors:** Maria Salnitska, Alexey Solodovnikov, Igor Orlov

**Affiliations:** 1 The Institute of Environmental and Agricultural Biology (X-BIO), University of Tyumen, Tyumen, Russia The Institute of Environmental and Agricultural Biology (X-BIO), University of Tyumen Tyumen Russia; 2 Natural History Museum of Denmark, Copenhagen, Denmark Natural History Museum of Denmark Copenhagen Denmark

**Keywords:** fauna, systematics, phylogenomics, barcoding, species, expeditions, tissues, vouchers, collection management

## Abstract

Staphylinidae beetles form a major portion of terrestrial biodiversity globally and, in particular, in Northern Eurasia, a large area with a historically better known north temperate, subarctic and arctic biota. However, even here, rove beetles remain amongst the so-called “dark taxa” with a high fraction of taxonomically unknown lineage diversity. The propagation of DNA-based technologies in systematic entomology in recent decades has brought new opportunities for biodiversity exploration, true also for Staphylinidae. Simultaneously, new methods have revealed limitations of specimens sampled and curated by traditional practices, as existing legacy collections, whether institutional or private, unfortunately do not always qualify as a source of DNA-grade material. In addition, both legacy and newly-collected DNA-grade material of Staphylinidae remain highly biased towards Central Europe, a region with a traditionally well-developed scientific infrastructure and long-established culture for the maintenance of entomological collections. To increase the degree of biodiversity knowledge for our target organismal group across the globe, efficient sampling of DNA-grade material and, in particular, the development of comprehensive local collections in under-studied regions is highly desirable. To facilitate that, here we provide a practical guide for collecting and curation of Staphylinidae with a focus on capacity building for DNA-grade collections in Siberia and elsewhere in Northern Eurasia.

## Introduction

With more than 64 000 described species, Staphylinidae (Insecta, Coleoptera), the rove beetles, is the second largest family amongst the Animal Kingdom after weevils (*Betz et al. 2018*). Rove-beetles are known from all continents except Antarctica where they inhabit nearly all ground-based microhabitats in all global terrestrial biomes. One possible reason for such evolutionary radiation and high diversity is their mainly small and flexible body (on average 2–8 mm in length) due to short elytra. This body plan enables rove-beetles to exploit ground-based crevices very efficiently using diverse feeding and other biological strategies (Thayer 2016, *Parker 2017*).

**Figure 1.**
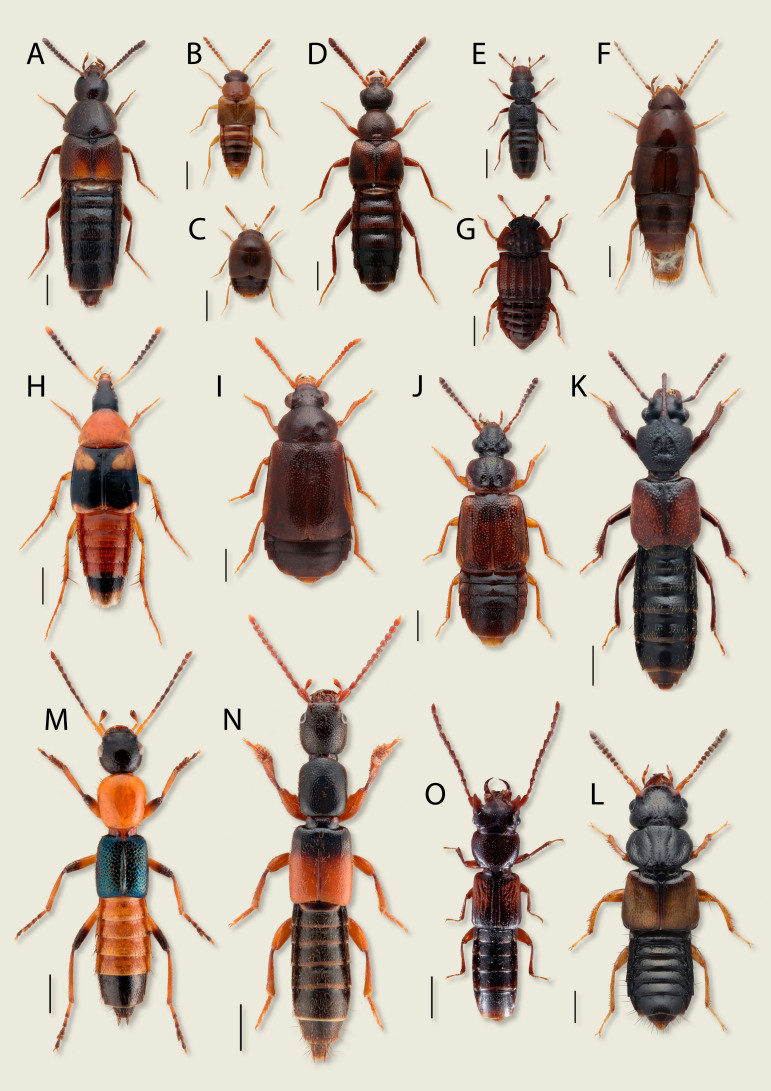
Diversity of Staphylinidae, some subfamilies in Northern Eurasia. **A** *Aleocharalaevigata* Gyllenhal, 1810 (Aleocharinae) 0.5 mm; **B** *Gyrophaenaaffinis* Mannerheim, 1830 (Aleocharinae) 0.5 mm; **C** *Cyphaseminulum* (Erichson, 1839) (Aleocharinae) 0.5 mm; **D** *Bolitocharaobliqua* Erichson, 1837 (Aleocharinae) 0.5 mm; **E** *Euaesthetusbipunctatus* (Ljungh, 1804) (Euaesthetinae) 0.5 mm; **F** *Habroceruscapillaricornis* (Gravenhorst, 1806) (Habrocerinae) 0.5 mm; **G** *Micropeplusfulvus* Erichson, 1840 (Micropeplinae) 0.5 mm; **H** *Lordithonlunulatus* (Linnaeus, 1760) (Mycetoporinae) 1 mm; **I** *Eusphalerumtenenbaumi* (Bernhauer, 1932) (Omaliinae) 0.5 mm; **J** *Omaliumrivulare* (Paykull, 1789) (Omaliinae) 0.5 mm; **K** *Blediusspectabilis* Kraatz, 1857 (Oxytelinae) 1 mm; **L** *Oxyteluslaqueatus* (Marsham, 1802) (Oxytelinae) 0.5 mm; **M** *Paederus riparius* (Linnaeus, 1758) (Paederinae) 1 mm; **N** *Lathrobiumelongatum* (Linnaeus, 1767) (Paederinae) 1 mm; **O** *Siagoniumquadricorne* Kirby, 1815 (Piestinae) 1 mm.

**Figure 2.**
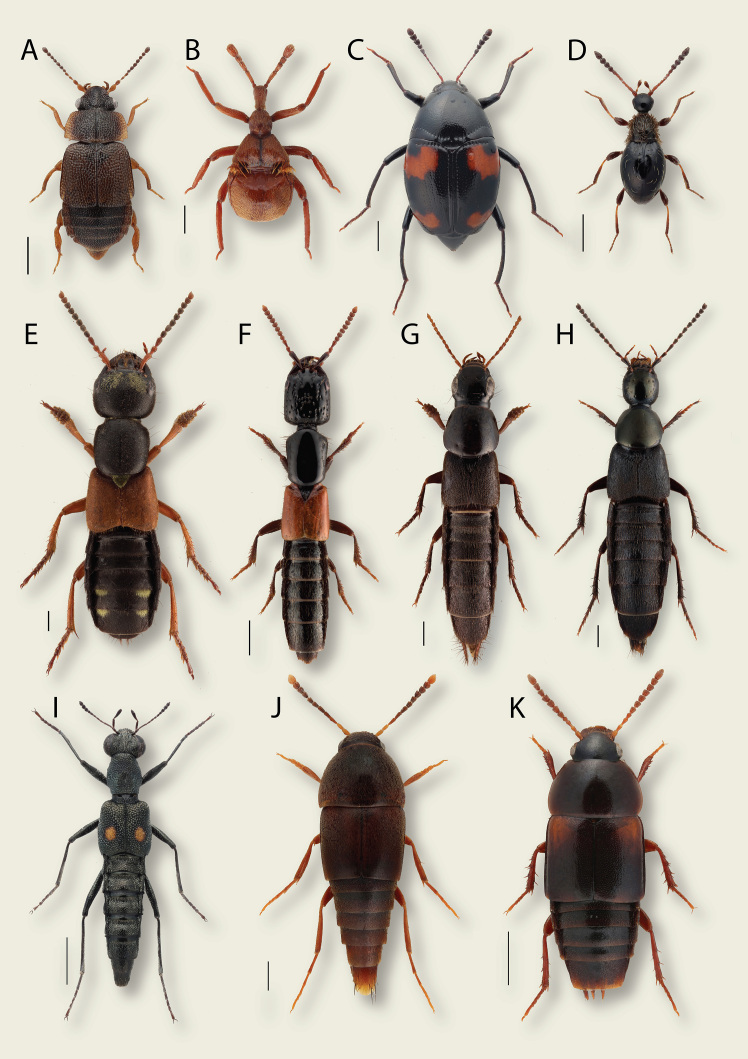
Diversity of Staphylinidae, some subfamilies in Northern Eurasia. **A** *Megarthrusdenticollis* (Beck, 1817) (Proteininae) 0.5 mm; **B** *Claviger longicornis* Müller, 1818 (Pselaphinae) 0.5 mm; **C** *Scaphidiumquadrimaculatum* Olivier, 1790 (Scaphidiinae) 1 mm (image by Anders Illum); **D** *Euconnushirticollis* (Illiger, 1798) (Scydmaeninae) 0.5 mm; **E** *Staphylinuserythropterus* Linnaeus, 1758 (Staphylininae) 1 mm; **F** *Gauropterusfulgidus* (Fabricius, 1787) (Staphylininae) 1 mm; **G** *Quediuslevicollis* (Brüllé, 1832) (Staphylininae) 1 mm; **H** *Philonthusdecorus* (Gravenhorst, 1802) (Staphylininae) 1 mm; **I** *Stenuscomma* Le Conte, 1863 (Steninae) 1 mm; **J** *Sepedophilustestaceus* (Fabricius, 1793) (Tachyporinae) 0.5 mm; **K** *Tachinusbipustulatus* (Fabricius, 1793) (Tachyporinae) 1 mm.

The great diversity and abundance of rove beetles (Figs 1, 2) make them a perfect proxy for investigation, comparison and monitoring of their entire habitats and ecological networks. At the same time, high taxonomic diversity of this group is the main limitation of its use in this capacity because the identification of the species or even a higher taxonomic category in Staphylinidae remains a complex task. Although scientific interest in Staphylinidae has significantly increased in recent decades, there are still numerous knowledge gaps about them, from poorly-explored faunas of entire continents to numerous taxonomic and phylogenetic problems. The poorly-known Staphylinidae fauna of the non-European territories of Northern Eurasia, mostly Russia and adjacent states, remains the most obvious knowledge gap as this large area is a seamless extension of the much better explored European region.

Scientific collections, institutional and private, general and specific, have always played a vital role in the process of the study of Staphylinidae. Originating as small noble collections in Europe and growing in the era of geographic discoveries in major European urban centres, currently, entomological collections constitute a vital part of public scientific infrastructure globally. Large sections of Staphylinidae form a part of any notable entomological collection today, not to mention the various specialised institutional or private collections of so-called amateur taxonomists. Despite some diversity of such collections, all of them are essentially assemblages of dry pinned specimens in entomological drawers filed in a cabinet. Seldom, they are complemented by more specialised types, for example, larval collections in 70% ethyl alcohol or bulk samples of unprocessed material kept in 70% ethyl alcohol, formaldehyde or dry on cotton layers awaiting sorting and mounting on insect pins.

The rise of molecular methods for the study of biodiversity during the last few decades has changed some approaches to the study of insects and, naturally, added new demands to the preservation and storage of material in collections in order to be DNA-grade (*Mandrioli 2008, Moreau et al. 2013*). In addition to extracting DNA from some of the legacy and recently-collected pinned specimens (Gilbert et al. 2007), specialised tissue collections kept under low or even ultralow temperatures have been developed to supplement traditional pinned collections in natural history museums (Vink et al. 2005, Marquina et al. 2021). As reviewed in Gusarov (2018), expansion of molecular methods has strongly impacted studies of Staphylinidae through large-scale molecular phylogenies (e.g. Elven et al. (2012), *Schomann and Solodovnikov (2017), Chani-Posse et al. (2018), Kypke et al. (2019), Żyła and Solodovnikov (2020), Brunke et al. (2021b)*), integrative taxonomy and population genetics (e.g. Song and Ahn (2014), *Brunke et al. (2020), Brunke et al. (2021a)*, Cai et al. (2021), *Salnitska and Solodovnikov (2021)*).

Molecular methods have revealed that classic collections of pinned Staphylinidae specimens are not always suitable for obtaining molecular data due to their collecting and storage conditions. Advances of phylogenetic and phylogeographic studies, dependent on a representative taxon sample, have also revealed how patchy and geographically biased available material is. In particular, Russia and adjacent countries, although covering major areas of Northern Eurasia, have a much thinner network of collections in contrast to the geographically smaller central and northern European countries. Even in well-sampled areas, legacy material, even if DNA-grade, may not be useful for detailed phylogeographic studies as often it comes with poorly-georeferenced locality data.

As an attempt to mitigate this bias, our paper provides guidelines on how to modernise existing collections or develop new ones to accelerate systematic studies of Staphylinidae in the target region using not only classic but and mainly so, modern molecular-based methods. It combines a succinct review of the currently available main literature and institutional resources which form the basis for building new collections, general recommendations and specific, practical tips for collecting, sorting and preserving Staphylinidae material, especially DNA-grade.

## Literature and other sources of published taxonomic information

The strategy of collecting material for a collection largely depends on the research tasks and projects, i.e. the short- and long-term goals. For example, whether one targets a comprehensive faunistic study of a particular region for Staphylinidae overall or searches for a certain taxon within limited or broad geographic areas for revisionary or phylogenetic work, will impact one's sampling approach. While Staphylinidae of Europe are better sampled, other territories of Northern Eurasia are unevenly studied, poorly or even barely touched by exploration. Sampling approaches for faunistic, taxonomic, phylogenetic, biogeographic or ecological studies will differ. The most valuable sources of information for planning any sampling effort or collection management are published specialised catalogues and comprehensive regional checklists, as well as taxonomic revisions and monographs. The amount of such literature is enormous and here we highlight a few of the most inclusive sources. The only recent printed World Catalogue of Staphylinidae (Herman 2001) is more than 20 years old. This catalogue is unique and very valuable as, in addition to the primary taxonomic information for each species, it traces all main taxonomic, faunistic and other publications, in which a given species was ever mentioned. Apart from being somewhat outdated, the Herman (2001) catalogue does not include six subfamilies, Aleocharinae, Paederinae, Pselaphinae, Scaphidiinae, Scydmaeninae and Silphinae. The first four subfamilies were not included for practical reasons or limitations and, at least for Pselaphinae and Scaphidiinae, this shortage is compensated by the world catalogues of these subfamilies (Newton and Chandler 1989, Löbl 2018), respectively. The subfamilies Scydmaeninae and Silphinae were included in Staphylinidae after the Herman (2001) catalogue was published. For Silphinae, there is a published catalogue of Nicrophorini (Sikes et al. 2002). For Scydmaeninae, there is a somewhat outdated published catalogue of genera (Newton and Franz 1998). The next rather inclusive and less outdated catalogue relevant for Northern Eurasia is that on the Palaearctic Staphylinidae (Schülke and Smetana 2015). It covers all subfamilies of Staphylinidae, except the most recently included Silphinae, but unlike Herman (2001), it contains only primary taxonomic information for species. Finally, the most complete, regularly updated and, thus, very valuable resource is the on-line world catalogue of Staphylinidae (Newton 2022) hosted by the Catalogue of Life (https://www.catalogueoflife.org/). Although there are many other, regional or taxonomically more restricted catalogues or checklist publications on Staphylinidae globally (e.g. Frank and Ahn (2011), Ahn et al. (2017), Asenjo et al. (2019), Li et al. (2019), Salnitska and Solodovnikov (2019)), the abovementioned three major catalogues form the most important reference sources for Northern Eurasian Staphylinidae, overall.

While the checklists and catalogues are the baseline resources, details must be sought in the main body of taxonomic literature, both legacy and recent. There is no single resource that would aggregate detailed information for all species of Staphylinidae for the entirety of Northern Eurasia. One of the most recent detailed resources is a volume covering several subfamilies of Staphylinidae for several European countries within the well-known German series on the beetles of Central Europe (Assing and Schülke 2012). Aleocharinae and some other smaller subfamilies (in their then-recognised status as separate families), however, are available only in the older edition of this series (Freude et al. 1974). The monograph of Staphylinidae of Western Palaearctic by Henri Coiffait (Coiffait 1972, Coiffait 1974, Coiffait 1978), the only other comparably large endeavour, has much broader geographic coverage, but it is much more outdated and does not cover all subfamilies of Staphylinidae either. In recent years, attempts have been made by Chinese researchers to produce national summary species-level monographs on Staphylinidae (Li et al. 2019), but they are far from complete too. Therefore, the only way of obtaining up-to-date species level information for Staphylinidae of Northern Eurasia is from primary taxonomic publications obtained via libraries, journal web sites, Biodiversity Heritage Library for legacy publications and other ways.

It is worth mentioning that the community of researchers studying Staphylinidae has accumulated all literature on the taxonomy and ecology of this group in one digital collection of PDF files, available for copying on a portable disc. The most updated PDF collection can be freely obtained from colleagues, for example, at the annual international meetings on biology and systematics of Staphylinidae, which have been held in May every year in Europe for more than 20 years. These meetings are an important venue to obtain information on all aspects of collecting Staphylinidae. Single references or any other bits of information can be obtained at any time by posting questions to the specialised listserver (https://sympa.uio.no/nhm.uio.no/info/staphlist) where the majority of contemporary colleagues with expertise on Staphylinidae are subscribed and can instantly provide help.

Taxonomic information on Staphylinidae, often supplemented by the high-resolution beetle images, is also available through a growing number of web resources, such as GBIF (https://www.gbif.org/), BeetleBase (http://beetlebase.com/), Danish Beetle Bank (https://danbiller.dk/), SCAN (https://scan-bugs.org/portal/), BioMap (https://baza.biomap.pl) and many others. However, coverage for Staphylinidae and the credibility of these online resources vary, often necessitating checking the primary resources from where these data came to these aggregators.

## Main Staphylinidae collections of the world relevant for Northern Eurasia

The most comprehensive collections of Staphylinidae with world coverage and a strong focus on Europe or Eurasia are concentrated in Europe, especially in the United Kingdom, countries of central Europe and Italy. Large legacy collections at the national natural history museums of London, Paris, Berlin, Vienna, Brussels, Geneva, Prague, Budapest and other European capitals, form the most crucial data banks for the taxonomic knowledge on Staphylinidae in Northern Eurasia. Collecting and collections of Staphylinidae are going strong in Japan, with the focus on the fauna of Japan itself. In addition, private collections of the prominent taxonomists working on Staphylinidae, mainly in Europe, most of which are bound by agreements to be integrated into institutional collections, are spectacular. Some institutional collections, not necessarily the largest, are bound to the active research programmes on Staphylinidae systematics or at least they are curated by the staff members with taxonomic expertise in this group (e.g. in Vienna and Geneva, Oslo, Wroclaw, Hamburg or Copenhagen-based natural history museums). A few institutions are particularly important for Staphylinidae systematics because of the large amount of type material. These are natural history museums in London (types of D. Sharp, M. Cameron, W. Kirby, T. Marsham and many others) and Vienna (types of O. Scheerpeltz, A. Horion, E. Eppelsheim, F. Wagner and others), the Royal Belgian Institute of Natural Science in Brussels (types of C. Fauvel, G. Fagel, A. Schuster and others), the Field Museum of Natural History in Chicago (USA) and the National Museum of Nature and Science (Japan). The Field Museum, in particular, is a depository for M. Bernhauer’s collection, one of the largest and most important legacy collections for Staphylinidae systematics. The National Museum of Nature and Science in Japan recently accessed the large collection of A. Smetana (*Smetana 2017*).

The Zoological Institute of the Russian Academy of Science, which has one of the largest zoological collections with a geographic focus on large areas poorly represented in other European collections, also has a notable collection of Staphylinidae. However, without specialised curatorial staff, this collection is difficult to navigate and a very large portion of it consists of poorly-georeferenced legacy material. Acceleration of taxonomic research on Staphylinidae in recent decades in China has prompted growth of respective collections there, especially in Beijing and Shanghai. Given that Russia and China occupy large areas of Northern Eurasia, the capacity of the Staphylinidae collections in those countries is still insufficient to support and promote the study of this mega-diverse taxon there. For example, in Russia, scientific institutions in Siberia or the Far East have collections that are an order of magnitude smaller compared to the collection in St. Petersburg, located in European Russia. Legacy material is predominating over recent accessions in the main institutional collection of Staphylinidae in Ukraine, Romania, Bulgaria and some other biodiversity-rich countries at the southern periphery of Northern Eurasia. Finally, large and biodiversity-rich areas of the Caucasus or Middle Asia do not have notable local collections of Staphylinidae at all. To promote the development of more collections in relevant local institutions, especially with a focus on desirable DNA-grade material, the following sections contain relevant guidelines.

## Collecting Staphylinidae

### Target habitats and microhabitats

Northern Eurasia comprises diverse landscapes (or biomes or habitats) from extensive tundras in the north to deserts in the south. Any habitat is a network of different microhabitats populated by certain species of Staphylinidae so that each collecting locality is represented by a microhabitat mosaic with different species communities. Thus, when the purpose of collecting is to study the entire fauna, it is necessary to cover all possible microhabitats within a locality by various collecting methods. If the target is to collect a certain taxon, it is necessary to focus sampling in its specific microhabitat and use the most efficient method. Overall, as staphylinids are mostly predators or saprophages in leaf litter and other humid decaying organic matters (Thayer 2016), their diversity and abundance are strongly correlated with the availability of such substrates. A smaller percentage of Staphylinidae are mycophagous and occur mainly in the fruiting bodies of fungi on the ground or tree trunks (Thayer 2016). Tree canopy or above-ground grassy vegetation are perhaps the least productive microhabitats for Staphylinidae, especially in Northern Eurasia. Most rove beetles that are found in vegetation climb the grasses or tree branches to facilitate their dispersal flight or to escape drowning in times of heavy rains or floods. (That is why sweeping grass vegetation above the water level in times of floods may be a very productive collecting method). However, some species occur in the vegetation more regularly because they are adapted for hunting there, for example, some *Stenus* (Fig. 2I) or *Paederus* (*Fig. 1*M) (Guseva and Shpanev 2019,Vijayaraghavendra et al. 2019). Finally, only a few groups occur in the vegetation because of their herbivory. For example, most of the adult *Eusphalerum* (*Fig. 1*I) (Omaliinae) species can be found on flowering plants where they feed on pollen, while *Trogophloeuspusillus* (Gravenhorst, 1802) (Oxytelinae) was reported to feed on fresh leaves and fruits (Tikhomirova 1973, Thayer 2016).

**Figure 3.**
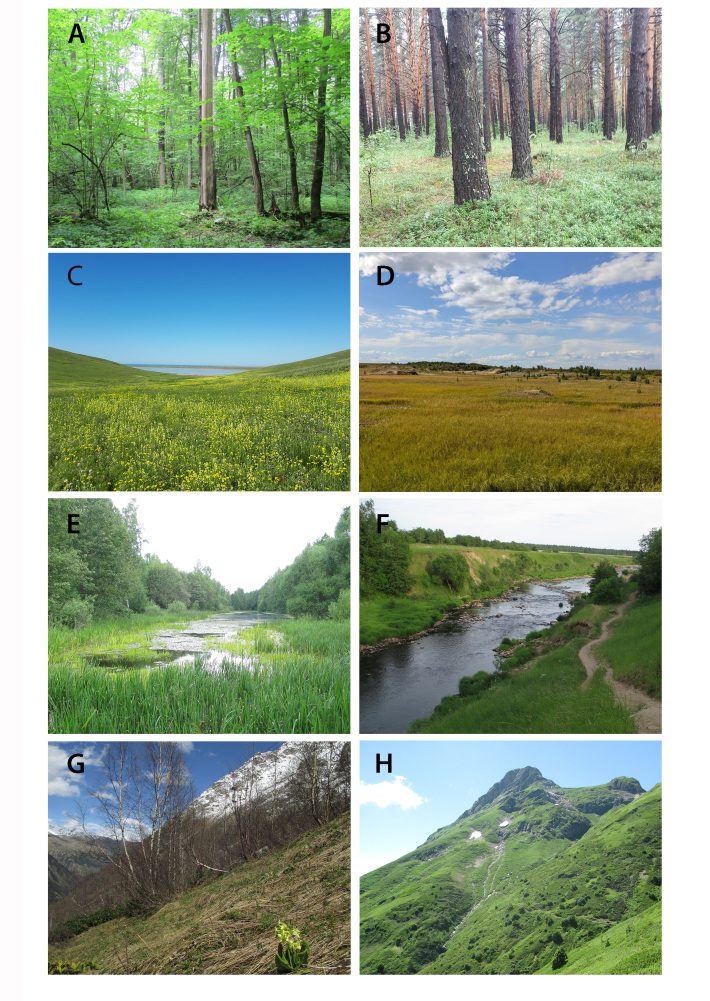
Main zonal and extra-zonal habitats for collecting Staphylinidae in Northern Eurasia. **A** broad-leaved forest (European Russia); **B** coniferous forest (European Russia); **C** steppe (European Russia); **D** tundra (West Siberia, Russia); **E** lake (West Siberia, Russia); **F** river; **G, H** subalpine zone in the mountains (Caucasus, Russia).

The most diverse community of species of Staphylinidae in Northern Eurasia is confined to the **forests**. For example, some of the largest genera, such as *Stenus* (*Fig. 2*I), *Lathrobium* (*Fig. 1*N), *Quedius* (*Fig. 2*G), *Tachinus* (*Fig. 2*K), *Mycetoporus* and many others are strongly associated with the Eurasian forest zone. The nemoral broad-leaved forests (*Fig. 3*A) are characterised by a larger biodiversity of Staphylinidae compared to coniferous forests (*Fig. 3*B). The **open zonal** (tundras, steppes, semi-deserts or deserts) (*Fig. 3*C, D) **or intrazonal** (meadows, bogs etc.) **landscapes** generally have less diverse species complexes of Staphylinidae compared to the forest communities. However, they accommodate some peculiar species and higher lineages. For example, many Paederinae or Oxytelinae are confined to open landscapes in the warmer areas of Northern Eurasia, or, on the contrary, some Omaliinae occur only in the tundra.

Regardless of the biome, rove beetles prefer humid microhabitats and are always abundant around **water bodies** such as **rivers, lakes, bogs, estuaries or sea shores** (E and F). There are many species and genera that are specialised to such wet habitats, for example, some *Stenus* (*Fig. 2*I), *Philonthus* (*Fig. 2*H), *Scopaeus*, *Bledius* (*Fig. 1*K), *Carpelimus*, *Falagria* and many others. Riverine forests and other near water habitats are often the main places to find Staphylinidae in dry biomes such as steppes. Some of these species are distinctly halophilous.

In addition, regardless of the biome, the **mountainous landscapes** (*Fig. 3*G, H) always form a more diverse and complex mosaic of habitats and microhabitats. The diversity and composition of montane faunas in Northern Eurasia depend on the geographic location and history of a given mountain system. Species communities may strongly differ from each other in different altitudinal zones of the same mountain system. In the more southern areas of Northern Eurasia, mountains harbour a high diversity of Staphylinidae, often with high rates of endemism.

**Figure 4.**
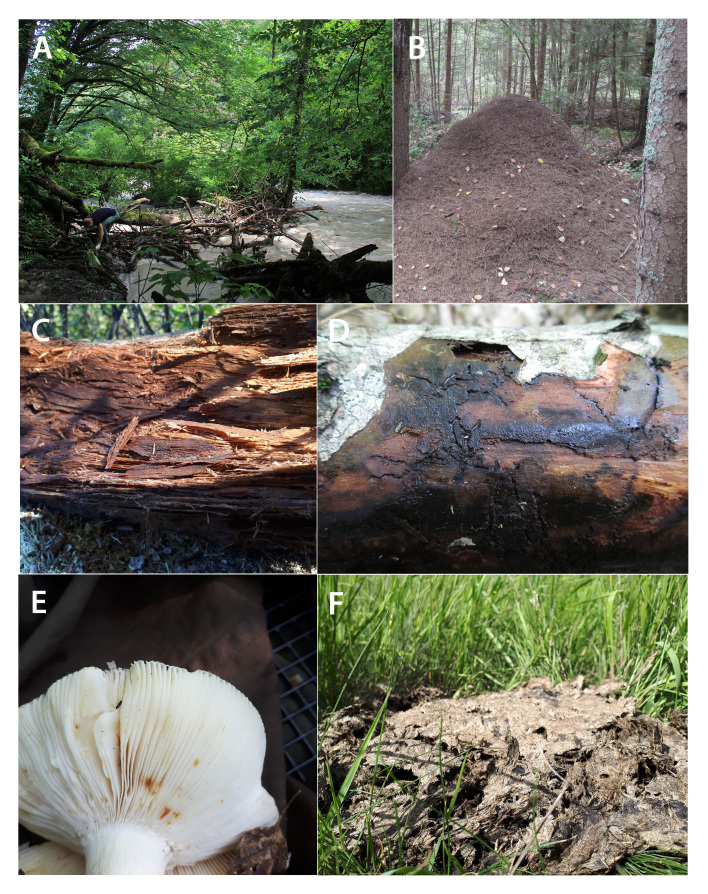
Main microhabitats for collecting Staphylinidae in Northern Eurasia. **A** river shore debris; **B** anthill; **C** rotten wood; **D** tree bark; **E** mushrooms; **F** cow dung.

Humid and fungusy thick **leaf litter** of mature broad-leaved forests is the most productive microhabitat (or substrate) for collecting rove beetles in Northern Eurasia. Patches of leaf litter with nearby piles of dead wood, growth of mosses or tree trunks with flowing sap are especially promising. Leaf litter of coniferous forests or drier forests or subalpine shrubs harbours less diverse species communities, but still is quite productive. Grassy leaf litter of the meadows, steppes, alpine grasslands or swamp tussocks is harder to process, but it is also an important microhabitat for Staphylinidae. Hydrophilous Staphylinidae are also often confined to the assemblages of wet leaf and other ground-based litter at the shores or splash-zones of various stagnant or running water bodies (*Fig. 4*A).

Despite the highly diverse species community associated with the water-edge habitats, there are no aquatic forms amongst rove beetles and, interestingly, only a few groups have evolved into specialised forms for life in the **deeper layers of soil** or in **caves** and other **subterranean cavities.** For example, **MSS** (Milieu Souterrain Superficiel according to Juberthie et al. (1980) and Mammola et al. (2016)) is a term describing a system of empty air-filled voids within rocky fragments, which is regarded as habitable for troglobiont Staphylinidae (Giachino and Vailati 2010). Many species that regularly occur in their usual habitats above ground surface, can be occasionally found in caves near entrances, especially in drier periods. Some riparian species dwell in gravel or dig deep in wet sandy or clay ground or they hunt openly by running on the soil surface of various types of shores.

Certain Staphylinidae species can be found in the **nests and burrows** of mammals and birds, where they mainly occur in the debris of the animal latrines. Some species, from time-to-time or exclusively, are found in the nests of social insects (Fig. 4B). For example, *Quediusdilatatus* (Fabricius, 1787) is closely associated with *Vespacrabro* nests, while a number of other rove beetles, especially from the subfamily Aleocharinae, are highly specialised social parasites in ant nests.

Staphylinidae are regularly found in **decaying wood or bark** (*Fig. 4*C, D), while some of them are highly specialised to these habitats. They occur in the fungusy and mossy crevices on the surface of logs or under and inside the decaying bark or, for some species, deeper inside the humid decaying wood itself. For example, all *Quedionuchus* species occur only under bark of broad-leaved and coniferous trees (Brunke et al. 2020) and *Hypnogyraangularis* (Ganglbauer, 1895), *Mycetoporuslepidus* (Gravenhorst, 1806), *Tachinusbipustulatus* (Fabricius, 1792) (*Fig. 2*K) and others are associated with decaying wood. There is a number of species, for example, *Hesperusrufipennis* Gravenhorst, 1802, a few species from the QuediussubgenusMicrosaurus and many Pselaphinae and Scydmaeninae, that are considered rare because they seem to be specific inhabitants of tree holes.

Many Staphylinidae, sometimes entire lineages, are more or less specialised to living inside the fruiting bodies of various types of **fungi** (*Fig. 4*E). Humid flesh of mushrooms has greater species assemblages than drier bracket fungi. Large and colourful species of *Oxyporus* are the most notable example of the highly specialised mycophagous Staphylinidae. Additionally, a mycobiont rove beetle community rapidly changes with the degree of fungal decay.

A significant number of Staphylinidae species are distinctly coprophilous and can be found in **faeces**, mainly of vertebrate animals. For example, moist cattle dung (*Fig. 4*F), especially after its first days of decay is quite rich for dung-inhabiting rove beetle species, especially *Philonthus* (*Fig. 2*H), *Aleochara* (*Fig. 1*A), *Oxytelus* (*Fig. 1*L) and others. A somewhat similar complex of necrophilous Staphylinidae can be found in **carrion**, i.e. mainly carcasses of the vertebrate animals. Some of the largest and most interesting looking species of Staphylinidae in Northern Eurasia, *Emushirtus* (Linnaeus, 1758) and *Creophilusmaxillosus* (Linnaeus, 1758), are examples of highly specialised coprophilous and necrophilous species, respectively. Nearly the entire subfamily Silphinae is specialised to necrophagy.

### Hand-collecting and trapping methods

**Figure 5.**
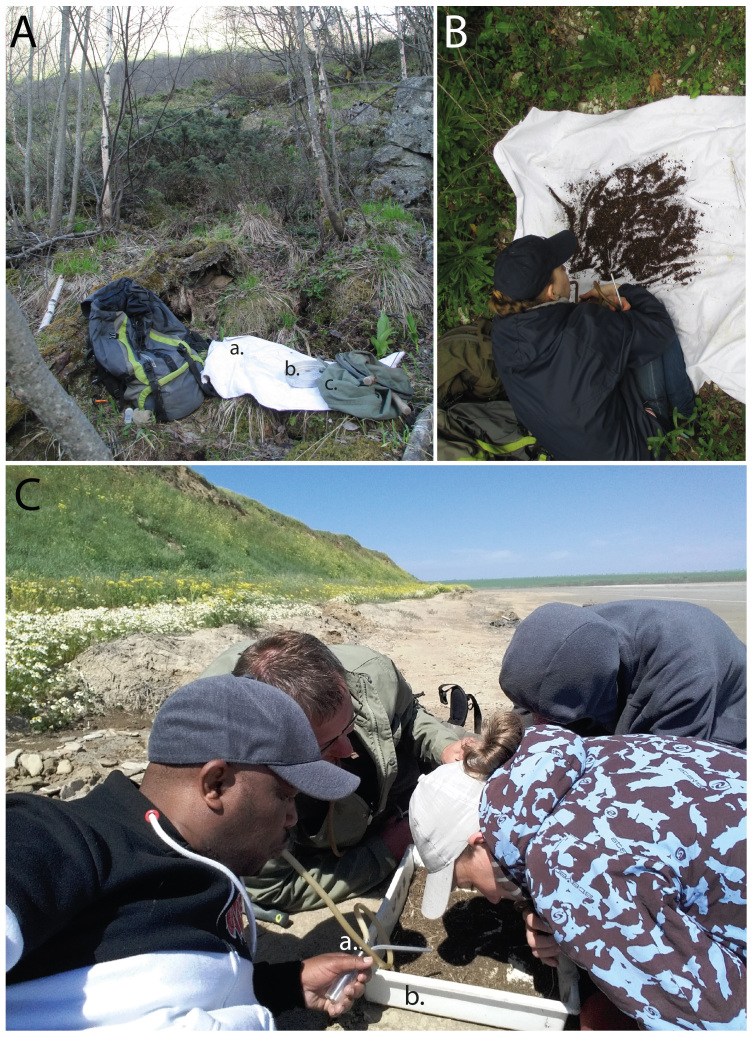
Sifting and processing of sifted debris. **A** basic tool kit for collecting: a white sheet, b geological sieve, c sifter; **B, C** processing of the debris: a aspirator, b plastic tray.

**Figure 6.**
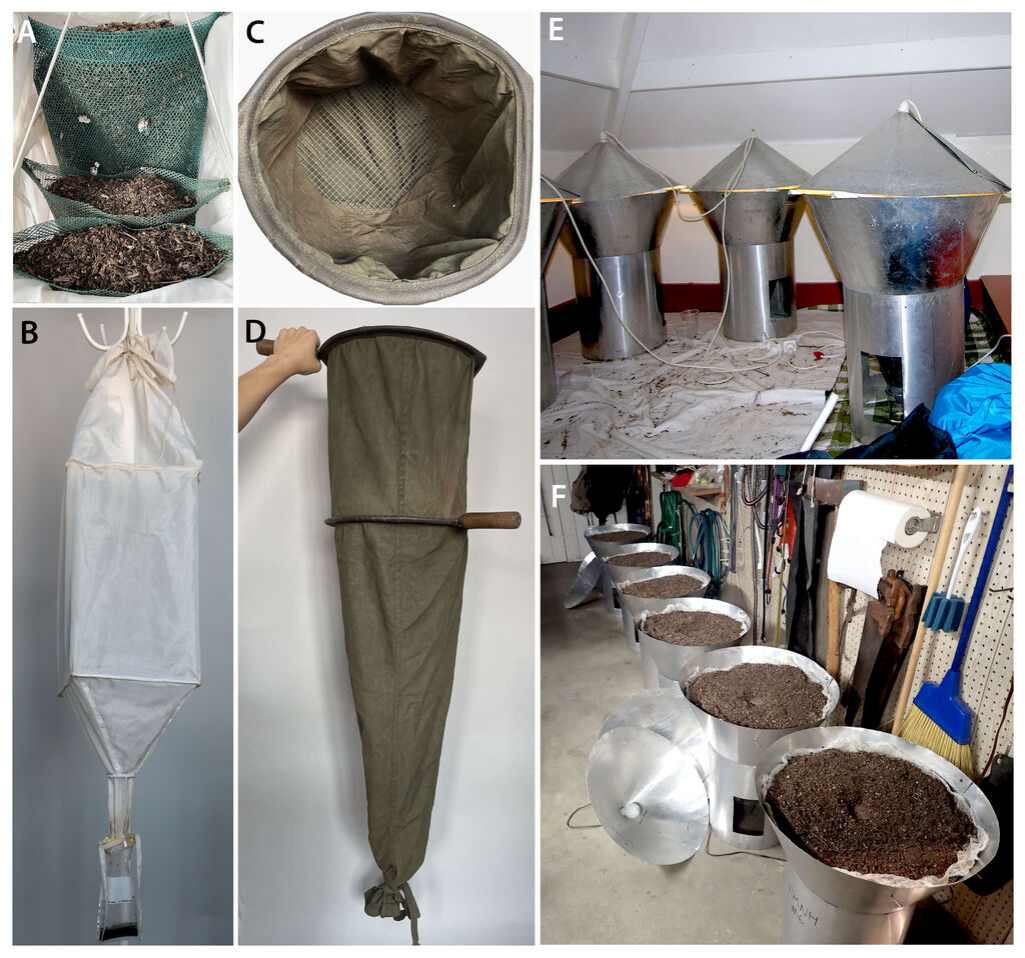
Winkler eclector. **A** eclector bags with portions of sifted debris inside; **B** general view of winkler. Sifter; **C** sifter mesh; **D** general view of sifter. Berlese funnels. **A** open, **B** closed.

Most Staphylinidae in the temperate or colder conditions of Northern Eurasia can be picked up by **hand-collecting** directly from the habitat they live in, as they rarely attempt to fly away quickly and none of Staphylinidae in that region is poisonous or otherwise hazardous to humans. Only a few large species from the subtribe Staphylinina may attempt to bite with their mandibles, which are, however, not strong enough to pose a significant threat. Having a collecting plastic tray and an aspirator or pooter (*Fig. 5*) allows one to manually process various substrates on a tray and efficiently pick up many staphylinids. Adding a knife, a small shovel or flathead screwdriver to this kit enables an entomologist to access spaces under bark or dig deeper into soil-based debris. The traditional and commonly-used insect net is rarely used by rove beetle collectors, as only a few Staphylinidae species are found in the vegetation and even those can be simply beaten into a tray. Instead of the net, a sieve or a sifter is the most useful, a 'must-have’ tool for collecting Staphylindae. Since the great majority of rove beetles are confined to various sorts of debris on the surface of the ground, **sifting** them out from those debris through various types of sieves is the most effective and universal collecting method. This could be done with simple geological sieves (*Fig. 5*A) or better with specialised entomological sifters (*Fig. 6*C, D). Layers of debris are actively hand collected (use robust garden gloves to avoid injury!) and piled into sieves to be shaken. Shaking triggers insects and other arthropods to move down through the mesh, which significantly reduces the amount of debris to be processed in search of rove beetles and the like. Normally sifters are the manual work for one person, but there is an attempt to invent a large high-throughput sifter (Grebennikov 2016). The sifted fraction can be manually checked for rove-beetles in the collecting spot in a tray or a large piece of cloth or plastic with a white surface to better notice moving beetles (*Fig. 5*). Hand collecting with the immediate checking of the smaller amounts of sifted material is great for learning where certain specimens are collected. However, such manual checks on the spot are time-consuming. Additionally, specimens that are slowly moving, stuck to wet debris or playing dead, especially in cold conditions, can be easily overlooked. Thereby, a variety of eclectors were designed for the complete processing of the sifted samples without human assistance, an essential tool for larger scale collecting efforts.

The **Winkler eclector** is the most universal as it is very portable and does not require electricity as a source of heat. It represents a long bag made from breathable white cloth internally sewed to two square rings, fastened on a top (*Fig. 6*B). A cup or a plastic bag (for example, Whirl-pack) with the preservation solution is attached to the exit opening of the eclector. Sifted leaf-litter or other debris is distributed into a few smaller bags of perforated cloth fixed inside an eclector by two hooks (*Fig. 6*A). This construction promotes drying of the leaf-litter, pressuring Staphylinidae and other invertebrates to move out and drop to the collecting cup or Whirl-pack. Usually, Winkler eclectors can be set up anywhere, but they are more effective in dry and warm circumstances, for example, when they are placed indoors at room temperature. Most Staphylinidae are rather fast to move out from sifted debris in Winkler eclectors after several hours (for example, overnight). However, some rove beetles and other invertebrate groups require more time for extraction as they can sit still in the bags, especially if conditions for the extraction are not perfectly warm and dry. This is especially true for small species, which can move to the centre of the perforated bag where the substrate remains moist. Extending the period of extraction for several days aided by more frequent periodical (e.g. twice a day) manual reloading (stirring) of the sifted debris will stimulate animals to move and to be extracted in such conditions.

The classical **Berlese funnel** eclector consists of a plastic or metallic cone (funnel) often with a mesh ring inside on which to place the sifted material. A cup with the preservation liquid is attached underneath the funnel, while the funnel is covered by a lid with an installed light bulb on its inner side. The funnel can be fixed on the ground in different ways, for example, by specially designed racks or simple plastic bucket depending on the funnel size. One of the best, most efficient and portable Berlese funnels we are aware of, are those used by Margaret Thayer and Alfred Newton (Fig. 6E, F). They are light and thin metal constructions that can be quickly assembled for use and disassembled for compact transportation from one collecting locality to another. The principle of extraction of the Berlese funnel is similar to the Winkler eclector, but with the drying process more efficient because it is accelerated by the lamp warming up sifted material. The light in the funnel must not be too powerful though, as the excessive heat may kill smaller beetles before they dig through the litter towards the bottom of the funnel. In addition, the use of Berlese funnels in field conditions is limited by the availability of a source of electricity.

Using a combination of several Winkler eclectors and Berlese funnels, one can efficiently process large amounts of sifted leaf litter and other debris in various conditions. During expeditions, the sifted material from various localities can be accumulated in breathable labelled canvas litter bags, which can be kept for several hours or even days until they are transported to suitable conditions for extraction.

**Figure 7.**
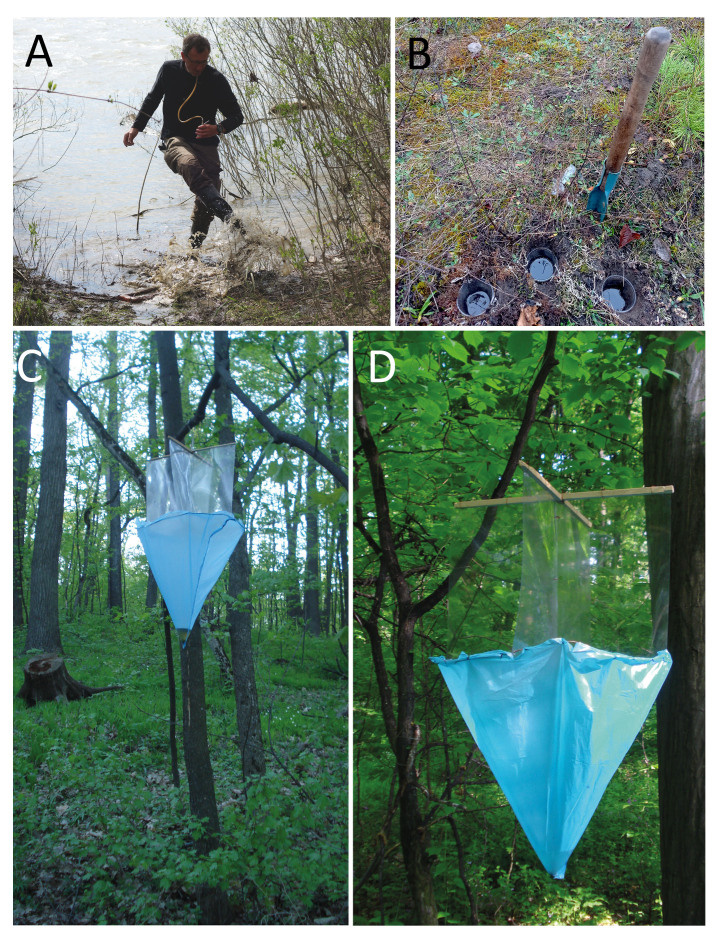
Collecting methods. **A** splashing; **B** pitfal traps. Window trap; **C, D** general view.

Unfortunately, not all kinds of debris are easy or possible to sift. For example, very wet moss or flood debris may be too sticky to be sifted. Additionally, liquified rotten mushrooms or dung or very fine-grained soil are difficult or pointless to sift. In those cases, piles of moss or similar substrates can be placed in the Berlese funnels directly. When this is not possible, for example, with soil or dung, a step of **flotation** may be added. For that, a target substrate is placed into a wide container with warm water to let Staphylinidae or other target animals float to the surface together with light organic debris. The latter material is collected by a small net, soaked on paper and afterwards placed for extraction of beetles into the eclectors. Using such flotation for large amounts of soil (so-called **soil washing**) was the only method that allowed us to discover diversity of the endogean subfamily Leptotyphlinae. Although the endogean fauna is mainly poor in Northern Eurasia due to the wiping out effect of Quaternary glaciation, many areas here are worth exploring by this method, described in detail in Andujar and Grebennikov (2021). Using the flotation principle is also helpful for hand-collecting of hydrophilic rove beetle communities concentrated at the river banks and similar water-edge habitats. **Splashing** water (Fig. 7A) on the shores triggers beetles to surface from litter, debris, moss or soil to avoid flooding, where it is easier to pick them. **Tramping** on the shores or treading with one’s feet on vegetation or other debris that is partly or entirely flooded, gives similar results.

Naturally, hand-collecting of rove beetles that occur in specialised, hidden habitats should be assisted by additional techniques and equipment. For example, caving skills and equipment are needed to explore caves for rove beetles or knowledge of ant and other animal biology and often digging equipment, are required to explore ant hills or underground animal nests. Occasionally a pyrethrum-based rapid killer insecticide (Raid or similar) may be used to spray fungusy or mossy logs to knock them out from crevices to a large piece of white cloth or plastic spread underneath (often referred to as small scale **fogging**). Interestingly, such fogging is often and efficiently used in tropical and south temperate conditions, while in north temperate zones, especially in Northern Eurasia, this method is not popular and, in our experience, not as efficient.

In addition to hand collecting aided by various extraction methods, there are a number of trap types used for collecting Staphylinidae. Traps bring non-stop long-term possibilities to sample rove beetles in the variety of habitats, but they demand time for installation and monitoring. Additionally, they non-selectively collect rove beetles along with any other similarly behaving invertebrates (and sometimes vertebrates, such as rodents and amphibians), so they produce mixed bulk samples that may demand labour-intensive subsequent sorting. Most trap types are the most effective when installed for longer periods of several days or weeks, which brings certain demands for the properties of preserving liquid used to kill and preserve collected organisms (for details see below).

**Pitfall traps** (Fig. 7B) are the most commonly used and easy type of traps that work on a principle that all active insects moving on a soil surface fall into a cup buried at ground level. If installed for longer periods of several days or weeks, a preserving liquid (best to use propylene glycol for DNA-grade material) must be added. In addition, the pitfalls must be covered by a roof to avoid rain water and to decrease the evaporation of preserving liquid. Easy-to-buy plastic party cups and plates may be used as pitfalls and roofs, respectively. Additionally, some specific attractants (dung, carrion, cheese etc.) can be placed nearby or attached near the entrance to the pitfalls with the aim of catching certain species. When a pitfall, unbaited or baited, is dug deeper in the ground, with or without a perforated pipe connecting the pitfall with the soil surface, it becomes a subterranean trap (Schmidt and Solar 2010, Pacheco and Vasconcelos 2012). Although pitfalls are the most frequently cited standardised method of sampling arthropods living on the soil surface (Querner and Bruckner 2010, Silva and Amaral 2013), it should be noted that, contrary to ground beetles (Carabidae) or spiders, it is not the best method for sampling the maximum diversity of Staphylinidae. Unlike ground beetles or spiders, many Staphylinidae are not actively running on the ground, but rather are dwelling within debris and, thus, are less prone to fall over the edge of a pitfall trap. That caveat notwithstanding, the pitfall is probably the only way to collect wingless, surface-active species efficiently. To target smaller rove beetles, which tend not to climb up when facing a barrier, it is very important to actively maintain the surrounding soil to be flush with the edge of the cup, not even slightly below.

Unlike pitfalls, **the flight intercept** or **window traps** (Chapman and Kinghorn 1955, Huizen 1980), which are traditionally used in the countries of Northern Eurasia much less than pitfalls, are in fact quite productive for sampling a diverse array of Coleoptera and Staphylinidae species, mainly in forests (Peck and Davies 1980) (*Fig. 7*C, D). Their success is based on the fact that many insects, including rove beetles, fly above the forest floor and fall to the ground when they collide with a vertical object. The flight intercept traps utilise this behaviour via a vertical screen of fine mesh or transparent glass or plastic, such as transparent kitchen film, stretched between two stakes just above a row of containers with preservative fluid (*Huizen 1980, Peck and Davies 1980*). A version with two crossed screens (Bouget et al. 2009) is more popular for catching saproxylic beetles and insects in general, but usually is less effective for Staphylinidae. There are more modifications of window traps, for example, a promising v-flight intercept trap version (Löbl et al. 2021) . Window traps work best on a long-term basis and yield surprising catches of insects not normally caught by other methods. Similarly to pitfalls, they should be equipped with a rain cover and filled with preservative fluid with low evaporation rate and ability to preserve DNA, like propylene glycol.

**Figure 8.**
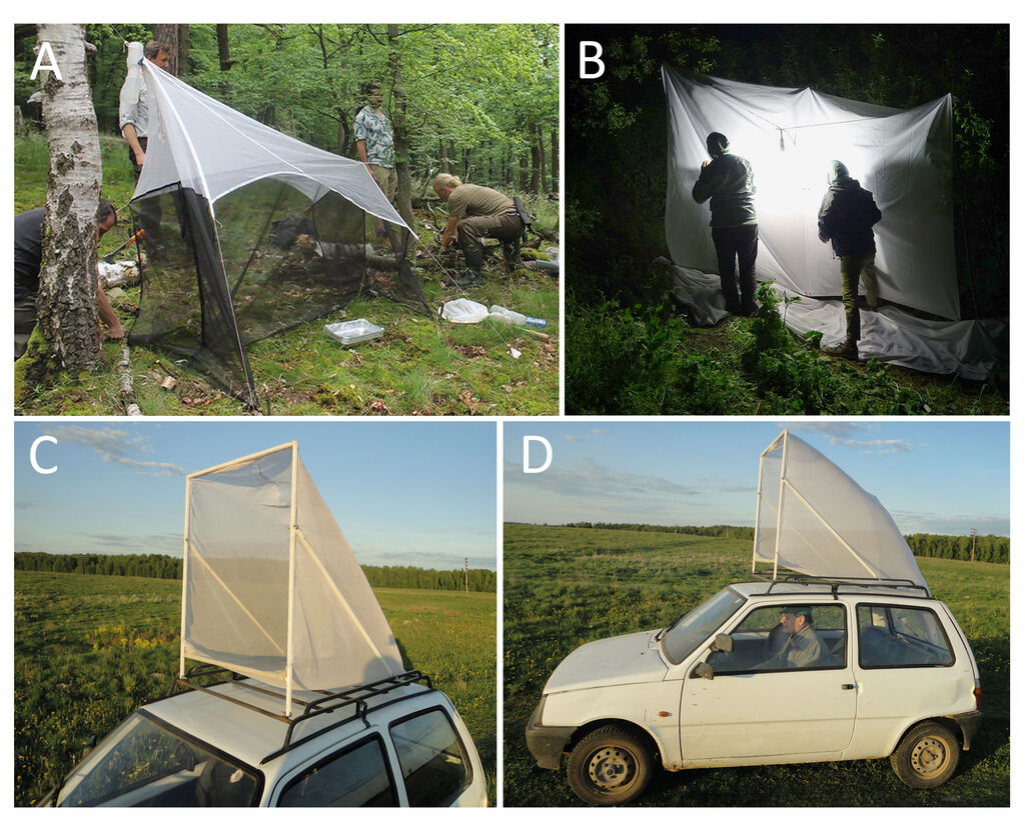
Trapping methods. **A** Malaise trap; **B** light trap. Car net; **C, D** general view.

The somewhat similar **Malaise traps** (Malaise 1937) are large, tent-like structures made of fine mesh netting that channel intercepted insects to a jar with preserving fluid mounted on its roof (*Fig. 8*A). They are one of the most widely used non-attractant, static insect traps (Muirhead-Thomson 1991, *Skvarla et al. 2021*) that also yield some Staphylinidae. They target flying insects, especially Diptera and Hymenoptera that climb up after encountering a barrier. The mainly ground-dwelling Staphylinidae, when encountering a barrier, more typically move down. However, Malaise traps may collect such species as well (Zilihona et al. 1998, *Achterberg 2009*). They can also bring those few arboreal Staphylinidae that are rarely collected by other methods. Unlike pitfalls or flight intercept traps, the collecting container of the Malaise trap has a small opening and, thus, it can retain 96% ethyl alcohol for sufficiently long to preserve DNA-grade specimens.

While the window and Malaise traps are static, a vehicle-mounted net (**car-net**) is a useful tool to actively sample flying Staphylinidae and other insects (*Fig. 8*C, D) (Jentzsch et al. 2017). The car-net was initially suggested by Freude et al. (1965) as a useful method for sampling beetles and since then it has been successfully used in various countries of Northern Eurasia (e.g. Kronblad and Lundberg (1978), *Rutanen and Muona (1982), Takahashi (1988)*). Car-nets are mounted on the roof or fender of the vehicle, about 1-3 m above ground level and collect insects in the special funnel from which they can be transferred to preserving liquid. It is assumed that the air in which the insects are flying is forced up and over the hood of the vehicle and into the net.

Finally, many night-flying insects, including some Staphylinidae, can be attracted to **light traps** (Fig. 8B). There is a great variety of light-trap constructions where attracted insects are funnelled to the vial with the preserving fluid or a killing agent. However, the simplest setup is a strong light bulb hung in front of a vertical white sheet with another white sheet spread on the ground underneath. The coming insects can be hand-picked directly from the vertical or horizontal sheets when they come to rest. An ultra-violet light bulb will increase the catch markedly (Ungureanu 1972, *Band et al. 2019*). It should be noted that use of light trapping for Staphylinidae in Northern Eurasia is limited to the southern areas only, where nights are long, dark and warm enough to support such night-flight activity. It is mainly rove beetle species living in the wet water-edge microhabitats that come to light. Most Staphylinidae never come to light.

As a concluding remark here, we stress that one should always stay open to new data about the biology of target groups, technological development and new inventions facilitating efficient collecting. For example, we plan to extensively try a powerful electrical vacuum cleaner to collect Staphylinidae from ground-based debris in Northern Eurasian grasslands, a method now becoming popular and productive in Central Europe.

### Preserving liquids for field collecting and bulk sample storage

Traditionally, rove beetle specimens collected in the field are killed and then preserved to be pinned (or point- or card-mounted on the insect pin). The main goal of pinning and drying a specimen on the pin is to preserve the exoskeleton intact for morphological examination. Most of the internal tissues decay and dry to some extent with such a curation technique. This is the main way to keep Staphylinidae in entomological collections, as with other Coleoptera, for decades to centuries. Due to the time lag between collecting and pin-mounting steps, there are a number of ways to keep specimens after they are killed and before they are mounted. One of the widely used methods is killing the material with and keeping it in 70% ethyl alcohol or in 70% alcohol with a slight addition of acetic acid. This solution keeps the specimens soft enough to be easily mounted after decades of storage. Another common way is to kill specimens with ethyl acetate and either keeping them in the same killing vials with sawdust or filter paper in the freezer before mounting or spreading them dry on layers of cotton. The latter method is especially widespread in Russia.

New requirements for material to be DNA-grade changed this practice. Now the purpose of the mainstream preservation is not only to keep a beetle body “intact” due to well-preserved sclerites of the exoskeleton connected by half-decayed, half-dried muscles and membranes, but to preserve its DNA for as long as possible.

Strategies for preservation of DNA and maintaining specimen qualities for easy mounting on insect pins do not always align (Andersen and Mills 2012), as the main target of the former is to block enzymes from destroying DNA and the main concern of the latter is to keep a specimen soft to nicely position its appendages during mounting. Additionally, a preserving method should be easy enough to be implemented in field conditions. Although detailed reviews of DNA preservation and long-term storage methods applicable for the entomological specimens have been made at various times (Prendini et al. 2002, Nagy 2010; Tan et al. 2021), many questions in this field remain open (Tan et al. 2021). In addition, preservation techniques widely practised by entomologists do not always account for all available knowledge about DNA preservation, but rather they are based on repeating somebody’s experience that is easy enough and showed some success. Another issue is that the DNA extraction and sequencing techniques are fast evolving towards their ability to cope with the degraded DNA, i.e. with the suboptimally preserved or simply traditionally pinned specimens that initially were not even intended for molecular work (Nakahama 2021; Lalonde and Marcus 2020; Mullin et al. 2022). According to the above-cited reviews and recent developments with museomics, preservation techniques may vary depending on the kind of the DNA for which data are sought. Another take-home message is that some of the expensive approaches, for example, immediate or long-term liquid nitrogen deep freezing of samples may not be necessary for the main goals of systematic entomology and related disciplines collections for which they are frequently used. As shown by Mullin et al. (2022) for traditional museum specimens, the initial post-mortem decline (fragmentation) of DNA is the strongest, after which a rate of DNA decay is low and consistent for decades. Finding a simple method for inhibiting this initial DNA decline is the most important target, which is beyond the scope of our experience and goals of this paper.

Killing by and preserving in 96% or even more highly concentrated ethyl alcohol is obviously a proven and simple method of inhibiting the initial post-mortem DNA degradation. It is important to understand that, to properly preserve a sample in 100% alcohol, it is necessary to dehydrate it by exchanging ethanol in the vial several times before storage. To facilitate that, the volume of alcohol should significantly exceed the volume of tissue, i.e. one should not fill the vial with specimens for more than ¼-⅓ of its volume. Specimens preserved that way may be mounted on insect pins afterwards or often, they are kept in absolute alcohol indefinitely, ideally at low (-20° – -80°C) temperatures. Some material meant for long-term DNA preservation for years and decades, if not centuries, is kept at ultralow temperatures in specialised cryo-tanks with liquid nitrogen (Rebecca et al. 1995, Mandrioli et al. 2006). Rarely, for high quality, full-genome sequencing, specimens are collected in liquid nitrogen directly. The problem with the mainstream, high concentration, alcohol-based preservation of DNA-grade material is dehydration of their muscle tissues which makes the specimens stiff in their death poses and hard to spread and mount properly for taxonomic study. Thus, either quality of the mounting is compromised by the DNA-grade priority or additional, often time-consuming softening procedures (Friedrich et al. 2014) are applied for specimens to be mounted from 96% alcohol. Occasionally, other chemical substances and methods are applied to preserve DNA- or RNA-grade specimens (for example, RNA later) (Gorokhova 2005), silica gel beads (Chase and Hills 1991, *Allison et al. 2021*), DESS (Yoder et al. 2006) and DETs (Frantzen et al. (1998) solutions and others).

As mentioned above, another challenge for DNA-grade preservation is posed by the long-term window and pitfall traps, where it is not possible to use high percentage alcohol as a preservation medium due to its high rate of evaporation. When these traps are not checked regularly, more viscous media like 100% (or as concentrated as possible) propylene glycol is used to preserve DNA-grade material. Moreover, unlike concentrated alcohol, propylene glycol does not make the specimens stiff and, as far as known (Nakamura et al. 2020; *Martoni et al. 2021*), 40%-100% propylene glycol preserves DNA as effectively as ethyl alcohol, though the higher the concentration, the better. Therefore, it is a promising preservation agent to consider not only for the traps, but also for long-term storage of Staphylinidae samples. If trap-collected material is to be stored in 96% alcohol, it can be easily transferred there from propylene glycol when traps are collected. Both liquids are mutually soluble.

### Collecting events and field labelling

**Figure 9.**
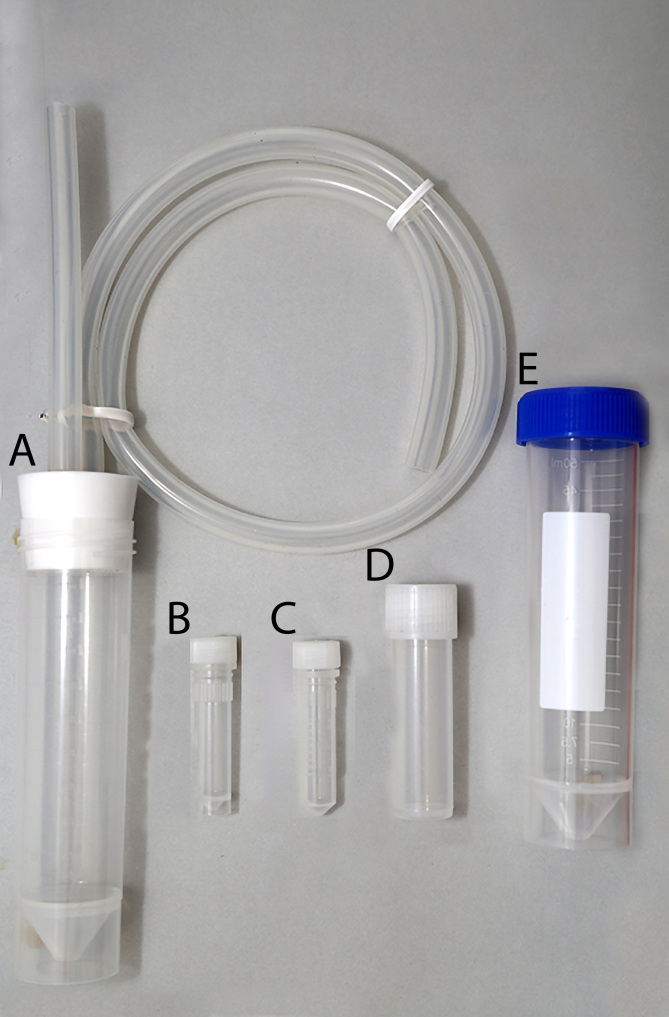
Aspirator and variety of vials. **A** general view of aspirator; **B, C** 2 ml; **D** 5 ml; **E** 50 ml falcon tube.

**Figure 10.**
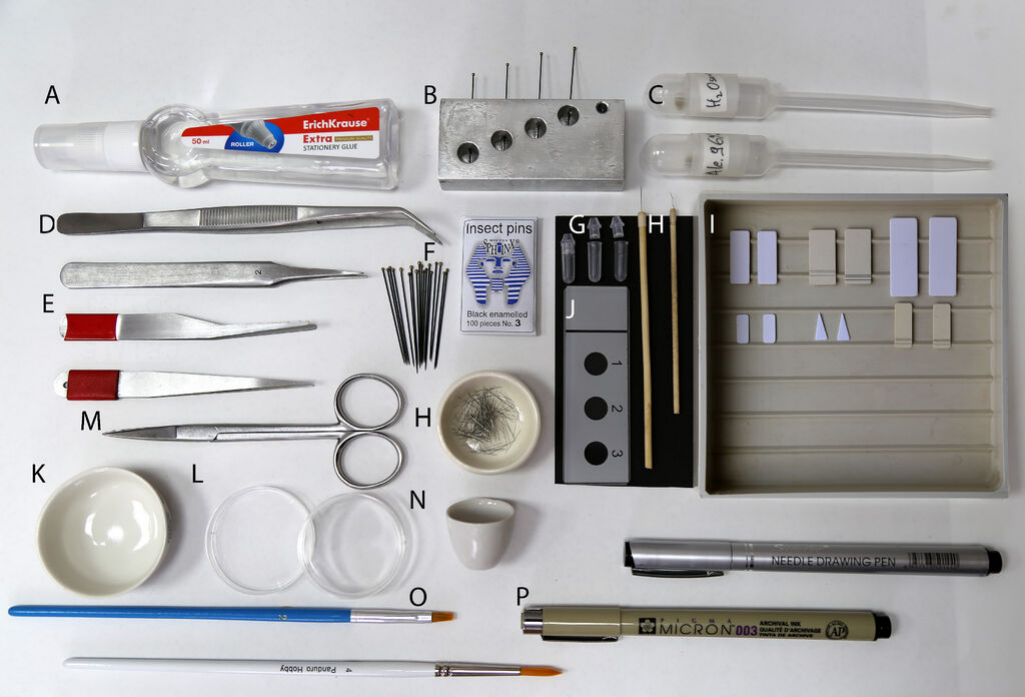
Basic entomological tools for mounting and dissecting specimens. **A** glue; **B** pinning block; **C** pipettes; **D** forceps; **E** soft forceps; **F** insect pins (No. 3); **G** genitalia vial; **H** minuten-based dissection tools; **I** mounting boards; **J, N** glass slides; **K** porcelain cup; **L** small Petri dishes; **M** scissors; **O** brushes; **P** alcohol-proof ink pens.

Regardless of the collecting technique, all samples from the same collecting event must be securely labelled in the field, to avoid any confusion or loss of information. A collecting event is an arbitrary concept, but usually this is a sample from the same trap collected during a certain period or a hand-collected sample from a microhabitat within a given locality taken on a certain date. Specimens hand-collected at the same collecting event are usually placed together in sizeable plastic vials (50 ml, for example, Falcon tubes) (*Fig. 9*E) with 96% ethyl alcohol for temporary storage during collecting. Bulk samples from traps may be voluminous and require even larger securely sealed plastic containers. In any case, any container should be supplied with a temporary label with the most essential information like geographic coordinates, date of collecting, habitat/microhabitat and collector information (see below). Even if all this information and more is written in a field log notebook where each collecting event is coded, the most essential data, especially geographic coordinates and date of collecting, must be duplicated to the field label in addition to a code corresponding to the field notebook entry. For fast and easy tracking of collecting events in the log notebook, amongst labelled samples or amongst those later recorded in a database, it is good to have the code system as easy and as logical as possible. For example, if collecting localities are denoted by numbers, collecting events corresponding to samples from various microhabitats within a given locality can be denoted by letters. Both primary labels and data in the field notebook should be written by pencil or alcohol-proof ink pen (for example, Sakura Pigma Micron liners, 0.1 and 0.5 mm) (*Fig. 10*P) to avoid the dissolution of the ink in alcohol or water.

### Short-term storage and transportation of samples from the field to laboratory conditions

For longer multi-day or week field trips, storage of samples in Falcon collecting vials or larger plastic containers is not practical, as they take significant space and have to be filled with alcohol up to the lid to avoid damage to specimens from shaking. Therefore, transferring them into securely sealed compact Whirl packs with a smaller amount of alcohol in a large plastic container that is kept away from day light and heat as much as possible is more practical. For air transportation, alcohol can be poured out from these bags completely for a few hours, to significantly reduce weight of the samples and comply with the safety regulations for luggage. Back in the laboratory, samples are transferred from the Whirl packs to collection cryo-vials or glass jars with fresh alcohol and printed finalised labels (for details, see below). Samples can be stored at room temperature for up to two weeks; for longer storage they should be deposited in a freezer at -20°C or colder.

### Labelling: principles and label content

**Figure 11.**
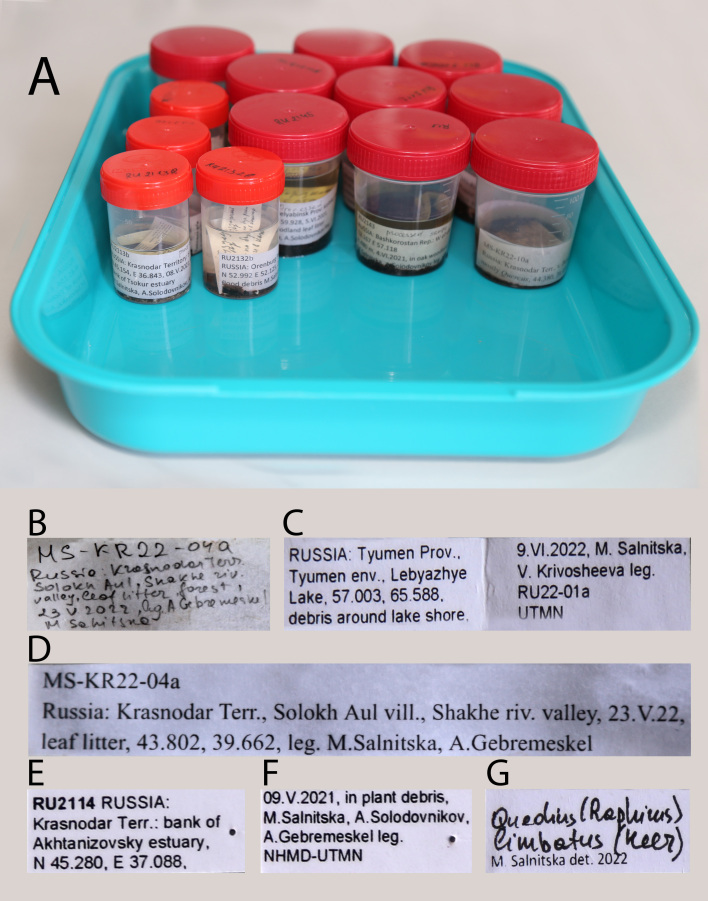
Examples of bulk sample and specimen labels. **A** temporary storage of bulk samples with the finalised geographic labels; **B** primary field hand-written labels; **C** finalised geographic labels for long term storage in 2 ml vials; **D** finalised geographic labels for temporary storage; **E, F** geographic labels for pinned specimens; **G** identification label.

**Figure 12.**
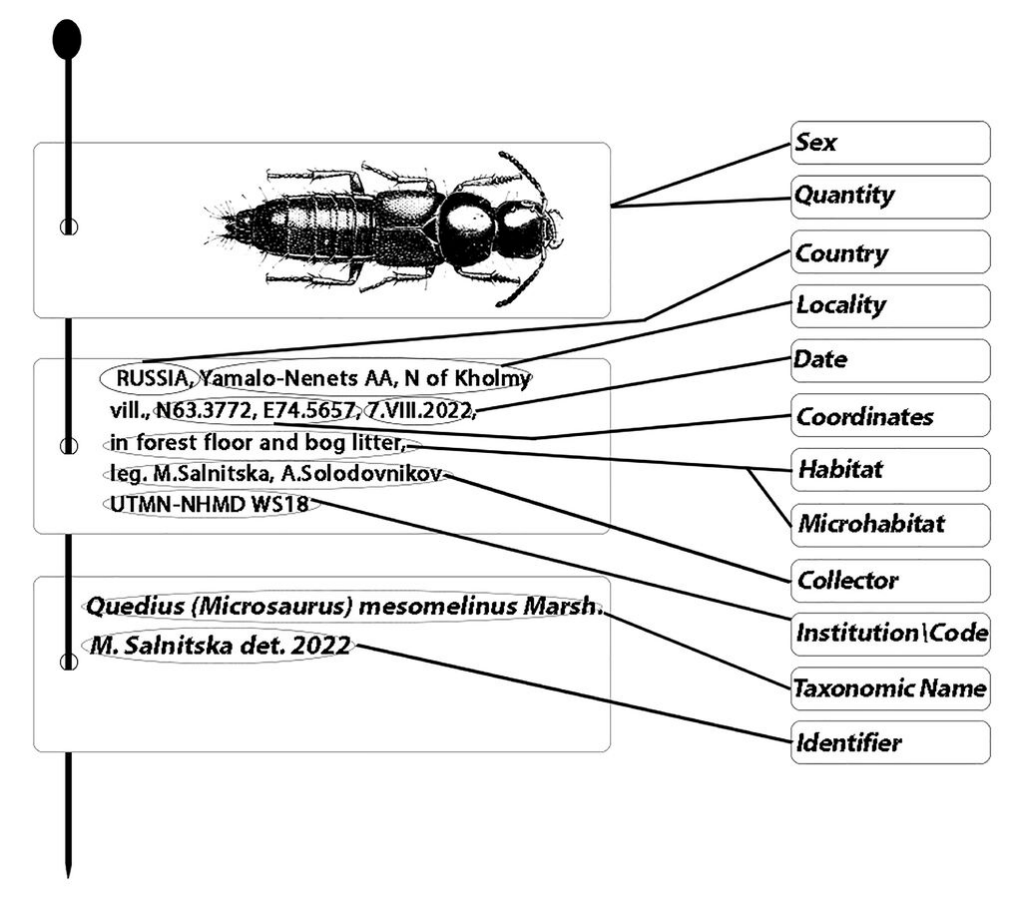
Necessary pieces of label information for a specimen to be properly prepared for the collection. Illustration of beetle from *Coiffait (1978)*.

Proper labelling is the most crucial condition for scientific material. The label must briefly, but unambiguously record the place, date and other conditions of a collecting event by having such information as country, province, locality, coordinates, habitat, microhabitat, date and collectors (*Fig. 11*B–G, *Fig. 12*). Even if a given collection is local, a label must be clear for the international scientific community and contain human-readable geographic information for a general idea about the location of a collecting event in addition to geographic coordinates and other numerical means for the computer-based retrieval of the geolocation and other information. The collecting date (or range of dates) must clearly denote the day (as arabic numerals) in the first position, month (as roman numerals) in the second position and a year of collecting in the last position (for example, 10.X.2010). Such a convention would prevent confusion caused by different national formats to denote dates. The collector’s name(s) and brief information about the habitat and microhabitat are other essential elements of the labels. Finally, the label may contain an abbreviation of the institution it belongs to and other internal codes for databases etc. (*Fig. 12*). Suggested optimal size for the pinned labels is 0.8 x 2 or 1 x 2 cm. If the data do not fit on one label, it can be split up into two labels pinned one under another to avoid very small font. Size and format for labels for wet collections depends on the size of vials (*Fig. 9*B–D).

## Dry and wet collections, curation process from bulk samples to individual DNA-grade specimens

In the countries of Northern Eurasia with a temperate climate, collections of Staphylinidae that serve as a source of DNA-grade material mostly comprise the following main types of material: (1) pinned adult specimens traditionally kept in drawers placed in cabinets at room or slightly cooler temperature, (2) wet material in 70% ethyl alcohol or other preservation liquids kept at room or slightly cooler temperature (bulk samples or individual adults or larvae awaiting to be sorted or mounted on pins) and 3) wet material in 96% ethyl alcohol permanently kept in freezers (usually bulk samples or individually sorted and identified adult or larval beetles). Dry pinned specimens may be DNA-grade to varying degrees, depending on the method of their collecting and preservation and their age (Gilbert et al. 2007, *Mitchell 2015*). Wet material in 70% alcohol or other liquids as a rule have the most degraded DNA. Material in 96% ethyl alcohol kept in freezers is mainly DNA-grade and meant to stay as such for the long term future. In some cases, derivatives from any of those specimens, for example, tissue samples in RNA later or other media or aliquots of extracted DNA or PCR products are also kept as separate units in the freezers or in liquid nitrogen cryo-facilities. Such derivatives are associated with their respective voucher specimens through a code system on their labels. In addition, in case of wet collections of the DNA-grade material, some representative specimens from the series (normally conspecific voucher specimens collected during the same collecting event) are pin-mounted and kept in the dry collection; a connection via indicative labels is maintained in such cases too. Upon arrival from the field site to the laboratory conditions, the fate of an individually collected specimen or a bulk sample of specimens may vary depending on the purpose or a research goal for which they were collected. An ideal scenario is that any species in the collection is represented at least by a series of identified specimens taken during the same collecting event, some of which are pin-mounted to become easily-accessed vouchers and some of which are kept in 96% alcohol under low temperatures as a long-term source of DNA-grade material. This ideal scenario requires the longest curatorial pathway (steps described below, plus databasing), which can be paused at various stages when more or less curated bulk samples are stored long-term.

### Storage of wet bulk samples

If the purpose is to keep mixed bulk samples in 96% alcohol under low temperatures long-term, the main standard to follow is proper labelling, using alcohol-proof thin (80 g/m^2^) paper (Southworth Paper, Hammermill, TerraSlate, BledProof and others) and ink (Canon, Xerox and others) and well-sealed quality jars or vials (Spectrum, Bormioli, IKEA and others).

### Morphosorting of wet bulk samples

**Figure 13.**
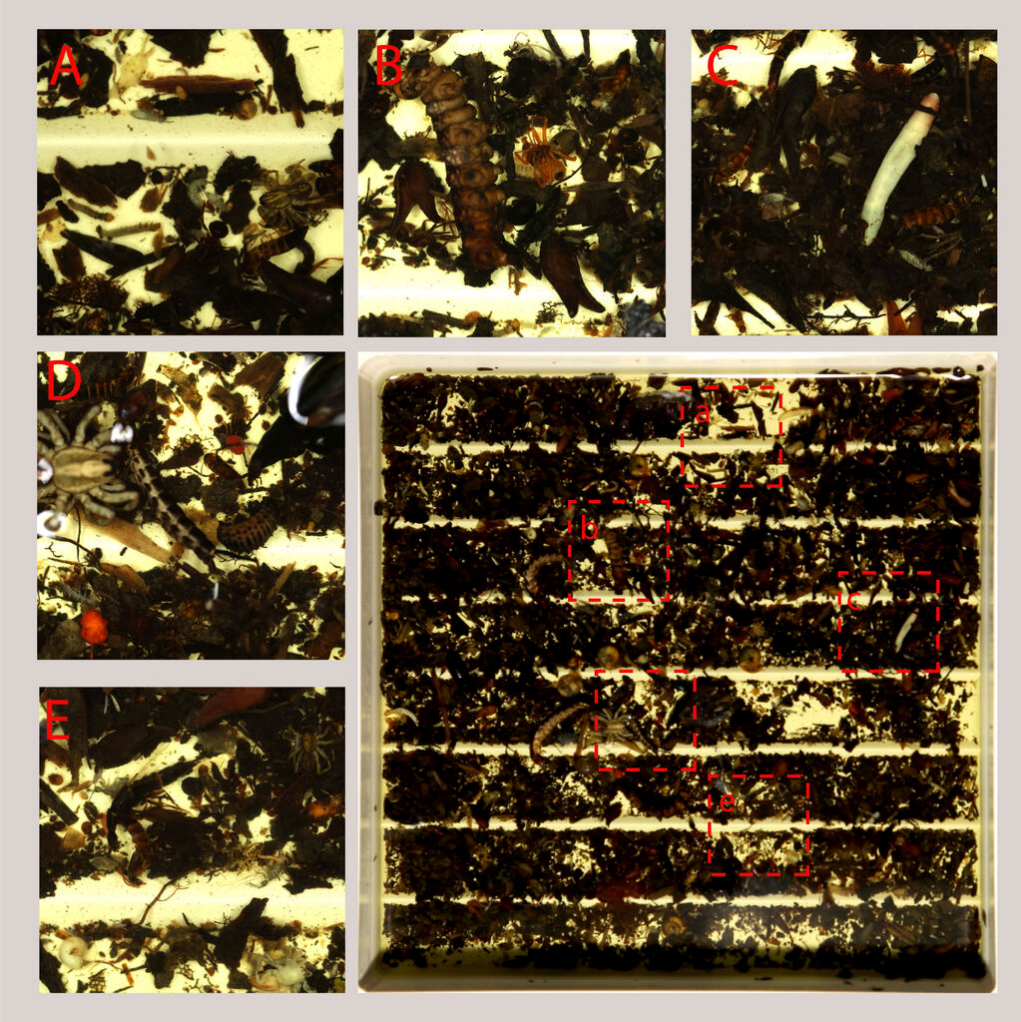
Sorting sifted leaf litter bulk sample, example of main groups of arthropods (and other invertebrates) to sort. **A** rove beetle, spider, springtail, ants; **B** spiders, larvae, ants; **C** rove beetles, springtails, annelids, aphids; **D** rove beetles, larvae, mites; **E** rove beetles, springtails, molluscs.

Morphosorting into certain taxonomic groups is the initial step of curating bulk samples for further, more detailed investigations. Morphosorting can be conducted at various taxonomic levels (for example, sort invertebrates into main classes, sort insects into orders, orders into families and so on) with the immediate aim to select target taxa (for example, family Staphylinidae) from the bulk sample (*Fig. 13*). Morphosorting of target taxa can be conducted down to the level of morphospecies, which is a universal term for the groups of specimens sorted and differentiated by characters of the external morphology using a binocular scope (Barratt et al. 2003, Ward and Stanley 2004). Although this method is not intended to consider taxonomic literature or taxonomic standards, some morphospecies might be a real taxonomic species or groups of closely related species. The accuracy of morphosorting depends on general characteristics of the target group, such as body size, intra- and inter-specific variability or quantity of the con- and heterospecific specimens to sort. The subsequent more thorough taxonomic study can adjust species limits by additional morphological or molecular characters. The morphosorting of Staphylinidae in alcohol allows quick preliminary division of the material into two categories: specimens which will be pin-mounted for dry collection and those to be kept in 96% alcohol at low temperature as DNA-grade material. As in alcohol-preserved material, the aedeagi are often protruded, some species can be identified more precisely already at this stage. Traditionally, morphosorting of Staphylinidae is performed with a binocular scope and general identification keys, such as Assing and Schülke (2012), Koszela et al. (2018) or others.

To efficiently perform morphosorting of a wet bulk sample, one must spread it (or its subset) on the bottom of a Petri dish or white sorting tray (*Fig. 13*) with alcohol using various forceps (*Fig. 10*D, E) and 3-5 ml pipettes (*Fig. 10*C). It is handy to use Petri dishes or sorting trays with the bottom subdivided into line sections, which allow better control of the checked versus unchecked sector of a sample under a binocular microscope. In the process of sorting, specimens of the same target morphogroup are placed together in one vial, each morphogroup going in separate 2 to 20 ml vials (*Fig. 9*B–D), depending on the quantity of material. Each vial is supplied with individual geographic and identification labels inside (*Fig. 11*E–G). If a bulk sample is rich and diverse and morphosorting is attempted down to a high detail, for example, a morphospecies level, it is practical to divide the process into steps and first sort a sample into orders, then isolate Staphylinidae, then sort them into subfamilies or other higher categories that finally are to be divided into morphospecies. Such an approach allows one to reduce the number of end-categories to trace at once (i.e. a number of individual vials corresponding to each category), which is demanding for concentration and may lead to misplacements of specimens in incorrect vials. Identification keys or other taxonomic resources including synoptic collections may be used at this preliminary identification step.

### Storage of wet individual samples for DNA grade cryo-collection

**Figure 14.**
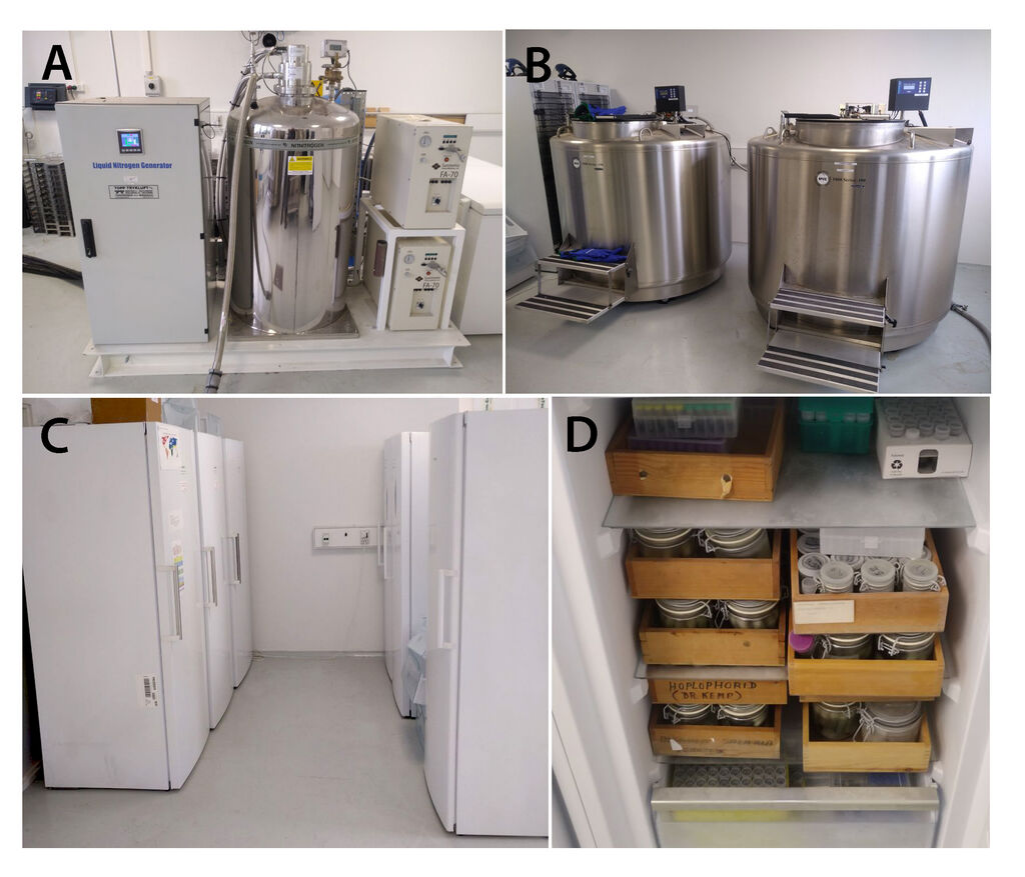
Example of infrastructure for a cryo-collection. **A, B** liquid nitrogen generator and tanks for storing samples under ultra-low (up to -80ºC) temperatures; **C** standing freezers for sample storage under low (-20ºC or lower) temperatures; **D** arrangement of jars and cryo-boxes with samples on shelves of a standing -20ºC freezer.

Individual wet samples in 96% alcohol after morphosorting are kept in properly labelled 2-20 ml cryo-vials (SSI, Eppendorf, Thermo FC, Corning, Accumax and others) (*Fig. 9*B–D) and organised in cryo-containers (Corning, Eppendorf, ThermoFS, Biologix and others) in freezers (Thermo Scientific, PHC, Eppendorf, Binder, Liebherr and others) (*Fig. 14*C). As in the case of wet bulk samples storage (see above), alcohol-proof paper and ink should be used for labelling individual vials. Each cryo-box and their groups (freezer shelves, if necessary) should be labelled as well, for example, with alcohol-proof markers, stickers or label pens (*Fig. 10*P). Unlike dry collections, where individual pin-mounted specimens are easy to visually inspect in the drawers or via examination of individual unit trays under a binocular scope, specimens in the cryo-collection are not so easily positioned to be seen through the semi-transparent plastic walls of cryo-vials. Therefore, curation of the cryo-collection is more of a challenge. As a response to that, in some cryo-collections, cryo-vials are organised not by taxonomic or otherwise meaningful criteria (for example geographically, by field trips etc.), but simply according to their accession order. In that case, vials are placed anywhere and provided with the individual codes entered in the respective database (for details, see about databasing below). A database query brings back a location usually coded by the shelf, box etc. number. Even though implementation of the taxonomic order in the collection requires more time and effort and repeated cycles of curation of the same vials, boxes or shelves when new accessions come or the classification changes, we still find it a more secure way to organise the cryo-collection rather than rely on the database alone for locating samples. Individual codes may indeed serve as an additional key to retrieve a location of an individual vial in the collection via a database, but the taxonomic system remains a safe navigator if, for any reason, the ‘blind’ data management goes wrong. As in the case of dry pinned collections (see below), a taxonomic system works best in combination with other systems (e.g. alphabetical or any other clear order of samples within major taxonomic categories). More information for easier visual navigation through the racks of cryo-vials can be provided by the colour-coded lids (*Fig. 14*C) (for example, to denote different regions).

### Mounting on insect pins

**Figure 15.**
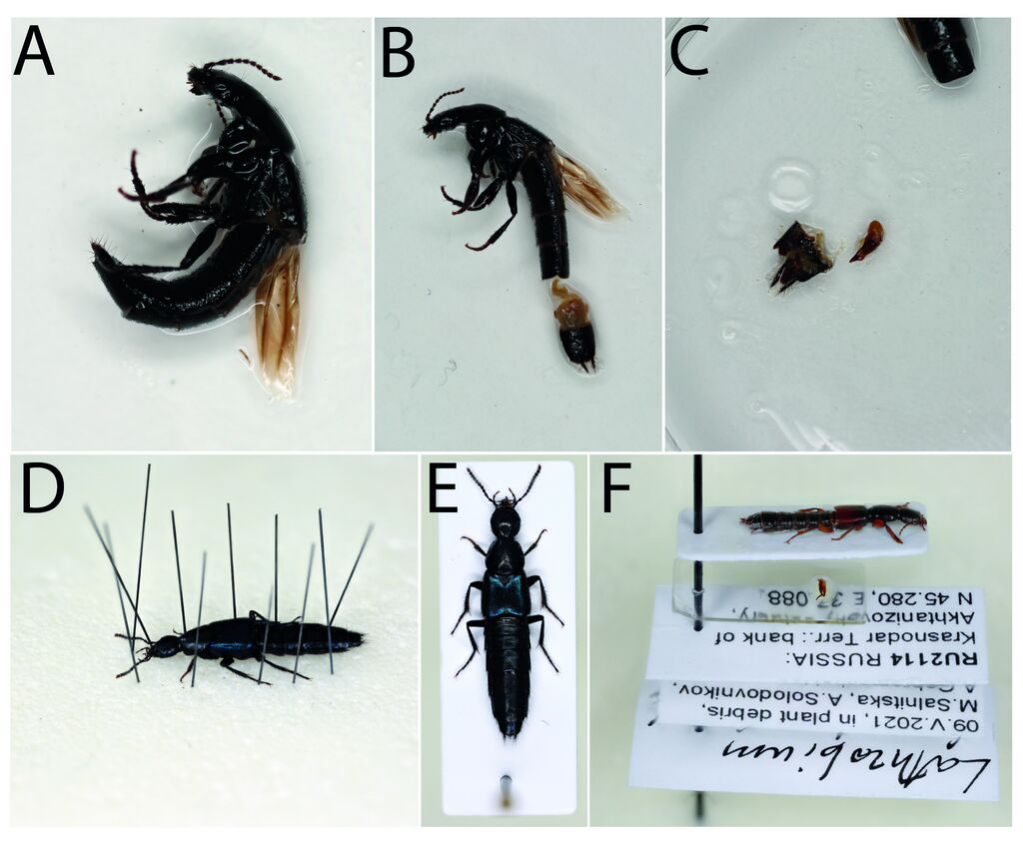
Mounting and dissection of specimens. **A, B, C** sequence of actions for the dissection of genitalia (for details see below); **D** fixation of the specimen in the necessary position; **E** properly mounted specimen; **F** example of the mounted, dissected and labelled specimen.

Traditionally, mounting large rove beetles on insect pins directly or, for smaller beetles, via gluing them to the card or point (*Fig. 15*E), has been the only main way of specimen preparation for collections. This method remains the main and most universal way to deposit specimens in a collection, especially given that now more and more prevailing NGS techniques reveal them to be DNA-grade at a higher rate than Sanger-based methods used in the past. There is a variety of mounting techniques (Schauff 2001, *Moghaddam et al. 2017*) (*Fig. 15*D). If a specimen is hardened after storage in alcohol or drying, it may be softened before mounting by soaking in water or in propylene glycol (Weigand et al. 2021). However, one must be aware that soaking in water may hinder DNA preservation. If a specimen is glued to card or point, a water-soluble glue (Herkules, EntomoAlex-gr, EntoSphinx, Erick Krause) (*Fig. 10*A) and others) is preferred over other glues (Canada balsam, casein-based glues, some PVA variations and others) (Deans 2018). Small Petri dishes (*Fig. 10*L), filter paper, pieces of foam, forceps (*Fig. 10*D, E), scissors (*Fig. 10*N), pins (*Fig. 10*F), brushes (*Fig. 10*O), minuten-based dissection tools (*Fig. 10*H), mounting boards (*Fig. 10*I) and mounting block (*Fig. 10*B) are used for mounting (*Fig. 15* E). Sturdy and sharp insect pins (stainless or enamelled pins, size 3 (diameter 0.5 mm, length 39 mm, for example, produced by EntoSphinx) (*Fig. 10*F) are used for mounting of all elements in the following order: a specimen, aedeagus or other preparation (if a specimen was dissected and preparation is mounted on a separate board), geographic label (or labels) and identification label (*Fig. 15*F). Labels for dry collections should be printed on a thick paper (from 160 to 230 g/m^2^).

### Mounting on insect pins with dissection of genitalia or other structures

Identification of a species of Staphylinidae often requires examination of the genitalia or terminalia. For that, these structures need to be dissected and prepared differently from the specimen. Occasionally, mouthparts or other structures are dissected too for closer examination. In all cases, it is best when dissected parts are mounted on the same pin with their respective specimen, usually in a special container (e.g. genitalia vial) (*Fig. 10*G) or on an extra card underneath (*Fig. 15*F). To dissect an aedeagus or terminalia, put a softened specimen in a small Petri dish (*Fig. 15*A), make an incision of connective tissues between 7^th^ and 8th segments of the abdomen using forceps or hooked pin, gently pull and separate the genital abdominal segments (8-10^th^ with the aedeagus inside) (*Fig. 15*B). Cut tissues between tergites and sternites of genital segments with a pin and forceps and gently pull out the aedeagus, cut muscles and connective tissues around the aedeagus, if necessary (*Fig. 15*C). If internal structures of an aedeagus are poorly visible due to the muscles inside, it can be boiled in a porcelain cup with 10% potassium hydroxide (KOH) (*Fig. 10*K, N) or kept there at room temperature from a few hours to overnight. After maceration, wash the aedeagus in the distilled water and place it into a genitalia vial (*Fig. 10*G) with glycerine or immerse it in a preparation medium like euparal, Cytoseal 60 (USA) or polyvinyl alcohol on a plate. The remaining abdominal segments can be glued to the card with a beetle or placed in the genitalia vial or mounted in the preparation medium next to the aedeagus; the latter is pinned underneath the specimen and above the first (geographic) label (*Fig. 15*F). Sometimes, large and hard-walled aedeagi of the larger species, especially those without taxonomically valuable internal structures to be seen through the aedeagus walls, are glued near the specimen on the same card. Extracting and preparing genitalia may also be done in the course of non-destructive DNA extraction. The dissected aedeagus or terminalia may provide sufficient tissue for DNA, while the proteinase/lysis buffer digestion process is a good way to clear genitalia and the DNA is a by-product. When the simultaneous DNA extraction and genitalia preparation are done in batches, the benefits may justify the costs.

### Organisation, storage and curation of specimens in dry collections

**Figure 16.**
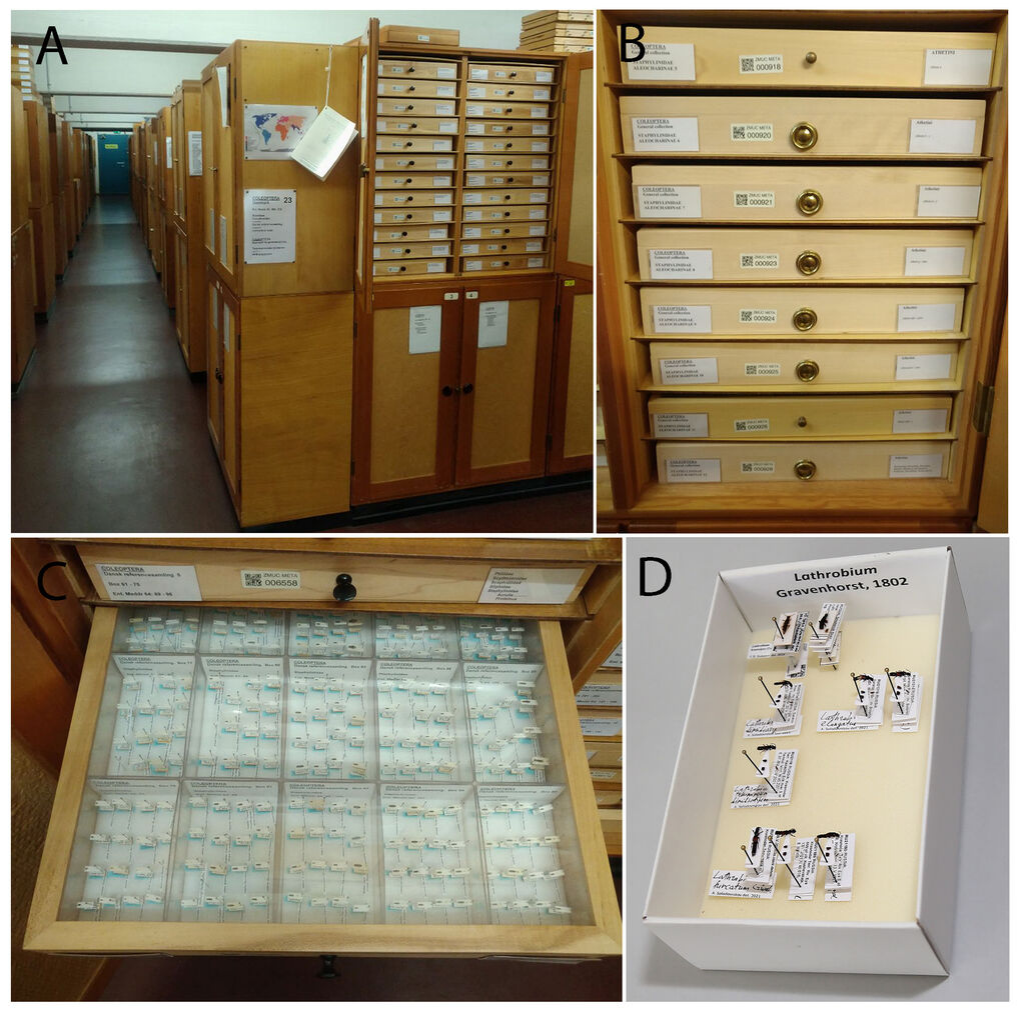
Example of the infrastructure for dry (pinned) collections. **A, B** wooden cabinets for entomological drawers, general view; **C** wooden entomological drawer with unit trays; **D** single unit tray, general view.

Despite some differences in collection organisation between institutions, the best and most accessible practice is to keep pinned Staphylinidae beetles in cardboard unit trays of two sizes (small and large) packed in wooden drawers (*Fig. 16*C, D) filed in wooden (*Fig. 16*A, B) or metal cabinets. Unit trays give much flexibility for curating the collection and they reduce the pace of possible spread of pest infection within and amongst the drawers. They compartmentalise different species, as specimens of the same species are placed in one unit tray (by rows running from the top left corner to the right bottom corner of a tray). Wooden drawers must have tightly-fitting lids to avoid infestation and dust. Glass lids are more practical than non-transparent wooden lids as they allow quick visual examination of whole drawers. The cabinets must have tightly-fitting doors, also to avoid infestation and dust. Ideally, a collection room with the cabinets must have climate control or at least maintain room or slightly lower temperature, have good ventilation and no direct sunlight. The cabinets, drawers and unit trays must be supplied with respective labels to facilitate quick navigation through the collection (*Fig. 16*A–C). It is practical to have the collection in alphabetic order; i.e. species within the genera, genera within tribes, tribes within subfamilies and subfamilies within the family Staphylinidae are arranged alphabetically. Undetermined material within a genus or higher taxonomic category is placed at the end, after the last determined (named) item. To avoid extensive recuration of drawers and cabinets when newly-accessed material is added or the collection is adjusted to taxonomic changes, it is good to budget as expansion space such at least one column of empty unit trays per drawer and at least a few empty shelves per cabinet. Sometimes, special markers are applied to the unit trays or drawers with the type or otherwise important specimens, for example, DNA-vouchers, or vouchers for the respective series of specimens in the wet cryo-collection etc. In other cases, smaller targeted synoptic collections can be arranged for special purposes within a larger general collection. For example, such synoptic collections may be regional or project-based collections where a male and female specimen per species are selected as examples for quick reference. Specimens in such collections may be mounted with special care (for example, spread nicely for photography) and arranged in any way that eases their quick comparisons.

## Databasing and digitalisation of collections

Modern advances in biological research that require processing information from publications, specimen meta-data, specimen images, DNA sequences and other large and diverse data are impossible without databasing and managing digital archives. Databases create a unified source of data for a certain project, geographic area or taxon. Databases are crucial for the digitisation of biological collections, a priority for the development of scientific infrastructure in recent decades (Blagoderov et al. 2012, *Schuh 2012, Tegelberg et al. 2014, Guénard et al. 2017, Hedrick et al. 2020, Popov et al. 2021*). There is a variety of commercial or free databasing software and data management systems for biodiversity sciences, for example, Arctos (http://arctosdb.org/), Arthropod Easy Capture (https://sourceforge.net/projects/arthropodeasy/), EMu (https://emu.axiell.com/), Papis (http://www.papis.dk/), SilverBiology (http://www.silverbiology.com/), Specify (https://www.sustain.specifysoftware.org/), Symbiota (https://symbiota.org/), TaxonWorks (https://taxonworks.org) and many others, not counting various local custom-made databases made with general software for relational database construction. Regardless of the system to be used for the digital support of a given collection of Staphylinidae or any collections-based project on this taxon, it should have a **systematic structure**, which as a rule constitutes a list of species (including synonyms, i.e. essentially a list of all available species-group names) and names for all the supra-specific systematic hierarchy. The next important input is the **geography** which is usually pre-populated along with the taxon-based data into the database. It is important to unify the design and composition of the geography data from the very first steps to avoid duplication and inconsistently filled data. In addition, it is necessary to unify the data on **bionomics** because usually, it can be described by a set of categories such as, for example, forest leaf litter, flood debris, dung and others. Additionally, forms for any data added in free format (verbatim data from labels, any free comments etc.) are essential. Some databases have ready-made and inflexible forms for data entry. Others, for example, EarthCape (Meyke 2019), a system used by the authors of this paper, allow the creation of custom environments for certain projects by a user without any special programming skills. EarthCape is a tool falling under the "back office" software category where the basic data visualisation, mapping, data outputs (tables, labels etc.) are tailored not only to research analyses, but also for managing logistics and communication when working on (data) papers (Meyke 2019). Choice of software for digital support of a collection is a complex topic beyond the scope of our paper and, to some extent, dependent on the institutional capacity and policy where a given collection of Staphylinidae would be developed.

## References

[B8170580] Achterberg K. (2009). Can Townes type Malaise traps be improved? Some recent developments. Entomologische Berichten.

[B8170589] Ahn K. J., Cho Y. B., Kim Y. H., Yoo I. S., Newton A. F. (2017). Checklist of the Staphylinidae (Coleoptera) in Korea. Journal of Asia-Pacific Biodiversity.

[B8170617] Allison M. J., Round J. M., Bergman L. C., Mirabzadeh A., Allen H., Weir A., Helbing C. C. (2021). The effect of silica desiccation under different storage conditions on filter-immobilized environmental DNA. BMC Research Notes.

[B8285716] Andersen J. C., Mills N. J. (2012). DNA Extraction from Museum Specimens of Parasitic Hymenoptera. PLoS ONE.

[B8283724] Andujar C., Grebennikov V. V. (2021). Endogean beetles (Coleoptera) of Madagascar: deep soil sampling and illustrated overview.. Zootaxa.

[B8170629] Asenjo A., Klimaszewski J., Chandler D. S., Fierros-López HE., Vieira J. S. (2019). Staphylinidae (Insecta: Coleoptera) in Latin America: synopsis, annotated catalog, diversity and distribution. Zootaxa.

[B8170639] Assing V, Schülke M (2012). Freude-Harde-Lohse-Klausnitzer - Die Käfer Mitteleuropas. Band 4. Staphylinidae I.

[B8170667] Band S. S., Sanjay V., Patidar S., Matcha N. (2019). Comparative efficiency of ultra violet black light lamp and mercury vapour lamp as a light source in light trap against major insect pest of Kharif crops. Journal of Entomology and Zoology Studies.

[B8170676] Barratt B. I.P., Derraik J. G.B., Rufaut C. G., Goodman A. J., Dickinson K. J.M. (2003). Morphospecies as a substitute for Coleoptera species identification, and the value of experience in improving accuracy. Journal of the Royal Society of New Zealand.

[B8170686] Betz O, Irmler U, Klimaszewski J (2018). Biology of rove beetles (Staphylinidae).

[B8170698] Blagoderov V., Kitching I. J., Livermore L., Simonsen T. J., Smith V. S. (2012). No specimen left behind: industrial scale digitization of natural history collections. ZooKeys.

[B8170708] Bouget C., Brustel H., Brin A., Valladares L. (2009). Evaluation of window flight traps for effectiveness at monitoring dead wood‐associated beetles: the effect of ethanol lure under contrasting environmental conditions. Agricultural and Forest Entomology.

[B8170747] Brunke A. J., Salnitska M., Hansen A. K., Zmudzinska A., Smetana A., Buffam J., Solodovnikov A. (2020). Are subcortical rove beetles truly Holarctic? An integrative taxonomic revision of north temperate *Quedionuchus* (Coleoptera: Staphylinidae: Staphylininae. Organisms Diversity & Evolution.

[B8170738] Brunke A. J., Pentinsaari M., Klimaszewski J. (2021). Integrative taxonomy of Nearctic and Palaearctic Aleocharinae: new species, synonymies, and records (Coleoptera, Staphylinidae. ZooKeys.

[B8170726] Brunke A. J., Hansen A. K., Salnitska M., Kypke J. L., Predeus A. V., Escalona H. (2021). The limits of Quediini at last (Staphylinidae: Staphylininae): a rove beetle mega‐radiation resolved by comprehensive sampling and anchored phylogenomics. Systematic Entomology.

[B8170759] Cai C., Tihelka E., Giacomelli M., Lawrence J. F., Slipinski A., Kundrata R., Donoghue P. C. (2021). Integrated phylogenomics and fossil data illuminate the evolution of beetles. BioRxiv.

[B8170771] Chani-Posse M. R., Brunke AJ., Chatzimanolis S., Schillhammer H., Solodovnikov A. (2018). Phylogeny of the hyper‐diverse rove beetle subtribe Philonthina with implications for classification of the tribe Staphylinini (Coleoptera: Staphylinidae. Cladistics.

[B8170790] Chapman J. A., Kinghorn J. M. (1955). Window flight traps for insects. The Canadian Entomologist.

[B8170799] Chase M. W., Hills H. H. (1991). Silica gel: an ideal material for field preservation of leaf samples for DNA studies. Taxon.

[B8170808] Coiffait H. (1972). Coléoptères Staphylinidae de la région Paléarctique occidentale. I. Généralités. Sous-familles: Xantholininae et Leptotyphlinae. Nouvelle Revue d’Entomologie Supplément.

[B8170826] Coiffait H. (1974). Coléoptères staphylinides de la région paléarctique occidentale II. Sous famille Staphylininae, Tribus Philonthini et Staphylinini. Nouvelle Revue d’Entomologie. Supplément.

[B8170835] Coiffait H. (1978). Coléoptères staphylinides de la région paléarctique occidentale III. Sous famille Staphylininae, Tribu Quediini. Sous famille Paederinae, Tribu Pinophilini. Nouvelle Revue d’Entomologie Supplément.

[B8170844] Deans A. R. (2018). A review of adhesives for entomotaxy. PeerJ.

[B8170863] Elven H., Bachmann L., Gusarov (2012). Molecular phylogeny of the Athetini-Lomechusini-Ecitocharini clade of aleocharine rove beetles (Insecta). Zoologica Scripta.

[B8170872] Frank J. H., Ahn K. J. (2011). Coastal Staphylinidae (Coleoptera): A worldwide checklist, biogeography and natural history. ZooKeys.

[B8170881] Frantzen M. A.J., Silk J. B., Ferguson J. W.H., Wayne R. K., Kohn A. M. (1998). Empirical evaluation of preservation methods for faecal DNA. Molecular Ecology.

[B8170940] Freude H., Harde K., Lohse G. (1965). Die Kafer Mitteleuropas. Band 1.

[B8170965] Freude H., Harde K. W., Lohse G. A. (1974). Die Käfer Mitteleuropas, Band 5, Staphylinidae II (Hypocyphtinae und Aleocharinae). Pselaphidae Goecke & Evers..

[B8170993] Friedrich F., Matsumura Y., Pohl H., Bai M., Hörnschemeyer T., Beutel R. G. (2014). Insect morphology in the age of phylogenomics: innovative techniques and its future role in systematics. Entomological Science.

[B8171048] Giachino P. M., Vailati D. (2010). The subterranean environment. Hypogean life, concepts and collecting techniques.

[B8171061] Gilbert M. T.P., Moore W., Melchior L., Worobey M. (2007). DNA extraction from dry museum beetles without conferring external morphological damage. PLOS One.

[B8171070] Gorokhova E. (2005). Effects of preservation and storage of microcrustaceans in RNAlater on RNA and DNA degradation. Limnology and Oceanography: Methods.

[B8171103] Guénard B., Weiser MD., Gomez K., Narula N., Economo EP. (2017). The Global Ant Biodiversity Informatics (GABI) database: synthesizing data on the geographic distribution of ant species (Hymenoptera: Formicidae). Myrmecological News/Osterreichische Gesellschaft fur Entomofaunistik.

[B8171136] Gusarov V. I., Betz O., Irmler U., Klimaszewski J. (2018). Biology of rove beetles (Staphylinidae).

[B8171149] Guseva O. G., Shpanev A. M. (2019). Rove beetles (Coleoptera: Staphylinidae) on agrolandscape herbaceous vegetation in the Leningrad Region. Russian Entomological Journal.

[B8171159] Hedrick B. P., Heberling J. M., Meineke E. K., Turner K. G., Grassa C. J., Park DS. (2020). Digitization and the future of natural history collections. BioScience.

[B8171172] Herman L. H. (2001). Catalog of the Staphylinidae (Insecta: Coleoptera). 1758 to the end of the second Millennium. I-VII (Parts 1-7).. Bulletin of the American Museum of Natural History.

[B8171780] Huizen T. H.P. Van. (1980). Why not use window traps for collecting Coleoptera and other flying insects?. Entomologische Berichten.

[B8171983] Jentzsch M., Glinka T., Link J., Lehmann B. (2017). Einsatz eines Autokeschers im Ziegelrodaer Forst-Ergebnisse und Bemerkungen zur Methode. Hercynia-Ökologie und Umwelt in Mitteleuropa.

[B8171190] Juberthie C., Delay B., Bouillon M. (1980). Sur l’existence d’un milieu souterrain superficiel en zone non calcaire. Comptes rendus de l'Académie des Sciences, Paris.

[B8285919] Koszela K., Żyła D., Solodovnikov A. (2018). The interactive digital key to rove beetles (Coleoptera: Staphylinidae) of Denmark.

[B8171199] Kronblad W., Lundberg S. (1978). Car catching: An interesting method for collecting beetles and other insects. Entomologiske Tidsskrift.

[B8171226] Kypke JL, Solodovnikov A., Brunke A. J., Yamamoto S, Żyła D (2019). The past and the present through phylogenetic analysis: the rove beetle tribe Othiini now and 99 Ma. Systematic Entomology.

[B8285813] Lalonde M. M., Marcus J. M. (2020). How old can we go? Evaluating the age limit for effective DNA recovery from historical insect specimens. Systematic Entomology.

[B8171257] Li L. Z., Hu J. Y., Peng Z., Tang L., Yin Z. W., Zhao M. J. (2019). Catalogue of Chinese Coleoptera..

[B8171291] Löbl I., Löbl I. (2018). World Catalogue of Insects 16..

[B8283756] Löbl I., Leschen R. A.B., Warner W. B. (2021). Scaphisomatini of Arizona (Coleoptera, Staphylinidae, Scaphidiinae) collected by V-flight intercept traps.. Revue suisse de Zoologie.

[B8171322] Malaise R. (1937). A new insect trap. Entomologische Tidskrift.

[B8171331] Mammola S., Giachino P. M., Piano E., Jones A., Barberis M., Badino G., Isaia M. (2016). Ecology and sampling techniques of an understudied subterranean habitat: the Milieu Souterrain Superficiel (MSS). The Science of Nature.

[B8171313] Mandrioli M., Borsatti F., Mola L. (2006). Factors affecting DNA preservation from museum‐collected lepidopteran specimens. Entomologia Experimentalis et Applicata.

[B8171304] Mandrioli M. (2008). Insect collections and DNA analyses: how to manage collections?. Museum Management and Curatorship.

[B8171354] Marquina D., Buczek M., Ronquist F., Łukasik P. (2021). The effect of ethanol concentration on the morphological and molecular preservation of insects for biodiversity studies. PeerJ.

[B8285861] Martoni F., Nogarotto E., Piper A. M., Mann R., Valenzuela I., Eow L., Blacket M. J. (2021). Propylene glycol and non-destructive DNA extractions enable preservation and isolation of insect and hosted bacterial DNA. Agriculture.

[B8171368] Meyke E. (2019). When data management meets project management. Biodiversity Information Science and Standards.

[B8171377] Mitchell A. (2015). Collecting in collections: a PCR strategy and primer set for DNA barcoding of decades‐old dried museum specimens. Molecular Ecology Resources.

[B8171386] Moghaddam M. H.G., Moghaddam M. G., Rakhshani E., Mokhtari A. (2017). An upgrade pinning block: a mechanical practical aid for fast labelling of the insect specimens. Biodiversity Data Journal.

[B8171404] Moreau C. S., Wray B. D., Czekanski-Moir J. E., Rubin B. E. (2013). DNA preservation: a test of commonly used preservatives for insects. Invertebrate Systematics.

[B8171413] Muirhead-Thomson R. C. (1991). Trap responses of flying insects. The influence of trap design on capture efficiency.

[B8285822] Mullin V. E., Stephen W., Arce A. N., Nash W., Raine C., Notton D. G., Barnes I. (2022). First large‐scale quantification study of DNA preservation in insects from natural history collections using genome‐wide sequencing. Methods in Ecology and Evolution.

[B8285736] Nagy Z. T. (2010). A hands-on overview of tissue preservation methods for molecular genetic analyses. Organisms Diversity & Evolution.

[B8285795] Nakahama N. (2021). Museum specimens: An overlooked and valuable material for conservation genetics. Ecological Research.

[B8285843] Nakamura S., Tamura S., Taki H., Shoda-Kagaya Etsuko (2020). Propylene glycol: a promising preservative for insects, comparable to ethanol, from trapping to DNA analysis. Entomologia Experimentalis et Applicata.

[B8171439] Newton A. F., Chandler D. S. (1989). World catalog of the genera of Pselaphidae (Coleoptera). Fieldiana Zoology.

[B8171448] Newton A. F., Franz H. (1998). World catalog of the genera of Scydmaenidae. Koleopterologische Rundschau.

[B8283691] Newton A. F. (2022). StaphBase: Staphyliniformia world catalog database (version Aug 2022). The Catalogue of Life, 2022.

[B8171493] Pacheco R., Vasconcelos H. L. (2012). Subterranean pitfall traps: is it worth including them in your ant sampling protocol?. Psyche.

[B8283531] Parker J. (2017). Staphylinids: quick guide. Current Biology.

[B8171533] Peck S. B., Davies A. E. (1980). Collecting small beetles with large-area "window" traps. The Coleopterists Bulletin.

[B8171511] Popov D., Roychoudhury P., Hardy H., Livermore L., Norris K. (2021). The value of digitising natural history collections. Research Ideas and Outcomes.

[B8286030] Prendini L., Hanner R., DeSalle R., DeSalle R., Giribet G., Wheeler W. (2002). Techniques in Molecular Systematics and Evolution. Methods and Tools in Biosciences and Medicine.

[B8171551] Querner P., Bruckner A. (2010). Combining pitfall traps and soil samples to collect Collembola for site scale biodiversity assessments. Applied Soil Ecology.

[B8171560] Rebecca A. R., Schwert Donald P., Ashworth Allan C. (1995). Field preservation of Coleoptera for molecular genetic analyses. Environmental Entomology.

[B8171569] Rutanen I., Muona J. (1982). Coleoptera collected with a car net in Finland. Notulae Entomologicae.

[B8171587] Salnitska M., Solodovnikov A. (2019). Rove beetles of the genus *Quedius* (Coleoptera, Staphylinidae) of Russia: a key to species and annotated catalogue. ZooKeys.

[B8171596] Salnitska M., Solodovnikov A. (2021). DNA barcode sheds light on species boundaries in the common morphologically variable rove beetle *Quediusumbrinus*-complex that puzzled taxonomists for more than a century (Coleoptera, Staphylinidae). Systematics and Biodiversity.

[B8171605] Schauff M. E. (2001). Collecting and preserving insects and mites. Techniques and Tools.

[B8171614] Schmidt F. A., Solar R. R.C. (2010). Hypogaeic pitfall traps: methodological advances and remarks to improve the sampling of a hidden ant fauna. Insectes Sociaux.

[B8171636] Schomann A. M., Solodovnikov A. (2017). Phylogenetic placement of the austral rove beetle genus *Hyperomma* triggers changes in classification of Paederinae (Coleoptera: Staphylinidae). Zoologica Scripta.

[B8171645] Schuh R. T. (2012). Integrating specimen databases and revisionary systematics. ZooKeys.

[B8171654] Schülke M., Smetana A., Löbl I., Löbl D. (2015). Catalogue of Palaearctic Coleoptera.Volume 2. Hydrophiloidea–Staphylinoidea, Revised and updated edition..

[B8171669] Sikes D. S., Madge R. B., Newton A. F. (2002). A catalog of the Nicrophorinae (Coleoptera: Silphidae) of the world. Zootaxa.

[B8171678] Silva L. N., Amaral A. A. (2013). Sampling of soil mesofauna and macrofauna with pitfall trap. Revista Verde de Agroecologia e Desenvolvimento Sustentável.

[B8171687] Skvarla J. M., Larson J. L., Fisher JR., Dowling A. P.G. (2021). A review of terrestrial and canopy Malaise traps. Annals of the Entomological Society of America.

[B8171696] Smetana A. (2017). Taxonomic review of the ‘quediine’ subtribes of Staphylinini (Coleoptera: Staphylinidae: Staphylininae) of mainland China. Nakladatelstvi Jan Farkač,.

[B8171709] Song J. H., Ahn K. J. (2014). Species delimitation in the *Aleocharafucicola* species complex (Coleoptera: Staphylinidae: Aleocharinae) and its phylogenetic relationships. Zoologica Scripta.

[B8171723] Takahashi K. (1988). Flight activity of insects sampled with a truck trap. I. Flight activity of Staphylinidae (Coleoptera). Konfyu.

[B8285786] Tan X., Ge L., Zhang T., Lu Z. (2021). Preservation of DNA for data storage. Russian Chemical Reviews.

[B8171732] Tegelberg R., Mononen T., Saarenmaa H. (2014). High‐performance digitization of natural history collections: automated imaging lines for herbarium and insect specimens. Taxon.

[B8283509] Thayer M. K., Beutel R. G., Leschen R. A.B. (2016). Handbook of Zoology. Coleoptera, Beetles..

[B8171750] Tikhomirova A. L. (1973). Morphoecological features and phylogeny of the Staphylinidae with a catalog of the fauna of the USSR. Nauka.

[B8171759] Ungureanu E. M. (1972). Methods for dissecting dry insects and insects preserved in fixative solutions or by refrigeration. Bulletin of the World Health Organization.

[B8171789] Vijayaraghavendra R., Lakshmi K. Vijaya, Chitra S., Malathi S., Jagadeeshwar R., Damodar C. H.R. (2019). Olfactory response of rove beetle, *Paederus fuscipes* (Curtis) to flower volatiles. Journal of Pharmacognosy and Phytochemistry.

[B8171804] Vink C. J., Thomas S. M., Paquin P., Hayashi C. Y., Hedin M. (2005). The effects of preservatives and temperatures on arachnid DNA. Invertebrate Systematics.

[B8285987] Ward D. F., Stanley M. C. (2004). The value of RTUs and parataxonomy versus taxonomic species. New Zealand Entomologist.

[B8171824] Weigand A. M., Desquiotz N., Weigand H., Szucsich N. (2021). Application of propylene glycol in DNA-based studies of invertebrates. Metabarcoding and Metagenomics.

[B8171833] Yoder M., De Ley I. T., King I. W., Mundo-Ocampo M., Mann J., M Blaxter, et a. l. (2006). DESS: a versatile solution for preserving morphology and extractable DNA of nematodes. Nematology.

[B8171846] Zilihona I., Heinonen J., Nummelin M. (1998). Arthropod diversity and abundance along the Kihansi Gorge (Kihansi River) in the southern Udzungwa Mountains, Tanzania. Journal of East African Natural History.

[B8171857] Żyła D., Solodovnikov A. (2020). Multilocus phylogeny defines a new classification of Staphylininae (Coleoptera, Staphylinidae), a rove beetle group with high lineage diversity. Systematic Entomology.

